# Prevalence of Behavioral and Psychological Symptoms of Dementia in Community-Dwelling Dementia Patients: A Systematic Review

**DOI:** 10.3389/fpsyt.2021.741059

**Published:** 2021-10-21

**Authors:** Chan-Young Kwon, Boram Lee

**Affiliations:** ^1^Department of Oriental Neuropsychiatry, Dong-eui University College of Korean Medicine, Busan, South Korea; ^2^Clinical Research Coordinating Team, Korea Institute of Oriental Medicine, Daejeon, South Korea

**Keywords:** dementia – Alzheimer's disease, behavioral symptoms, neurobehavioral manifestations, prevalence, systematic review

## Abstract

**Background:** Identifying the characteristics of behavioral and psychological symptoms of dementia (BPSD) associated with different dementia types may be a promising strategy to effectively deal with BPSD. We aimed to synthesize the prevalence rates of BPSD characteristics in community-dwelling dementia patients.

**Methods:** We searched Medline, EMBASE, and PsycARTICLES databases for original clinical studies published until December 2020 that enrolled at least 300 community-dwelling dementia patients. The methodological qualities of prevalence studies were assessed using the Joanna Briggs Institute's critical appraisal checklist.

**Results:** Thirty studies were included. The prevalence of the BPSD characteristic ranged from 4 (elation and mania) to 32% (apathy) in the pooled samples. The prevalence of delusions, anxiety, apathy, irritability, elation and mania, and aberrant motor behavior in Alzheimer's disease patients was 1.72–2.88 times greater than that in vascular dementia (VD) patients, while the prevalence of disinhibition in VD patients was 1.38 times greater. The prevalence of anxiety, irritability, and agitation and aggression, delusion, hallucinations, apathy, disinhibition, and aberrant motor behavior tended to increase as the severity of dementia increased, while that of depression, eating disorder, sleep disorders, and elation and mania tended to stable. In community-dwelling patients with dementia, the pooled prevalence of apathy, depression, anxiety, irritability, agitation and aggression, sleep disorders, and eating disorder was higher than 20%, while that of disinhibition and elation and mania was lower than 10%.

**Conclusion:** Overall, the pooled prevalence of apathy, depression, anxiety, irritability, agitation and aggression, sleep disorders, and eating disorder was generally high in patients with dementia. Also, the prevalence of some BPSD characteristics differed according to the type and the severity of dementia. The methodological quality of the included studies is not the best, and high heterogeneity may affect the certainty of the findings. However, the results of this review can deepen our understanding of the prevalence of BPSD.

**Systematic Review Registration:** https://osf.io/dmj7k, identifier: 10.17605/OSF.IO/DMJ7K.

## Introduction

Dementia is a common neurodegenerative disease in the elderly, causing a worldwide public health burden. Due to the growing aging population worldwide, the prevalence of dementia is increasing exponentially. According to the World Health Organization, the number of patients with dementia is expected to reach 115.4 million by 2050; however, this fact is largely ignored (1). Dementia is a syndrome that can be caused by various diseases, and among those, Alzheimer's disease (AD) and vascular dementia (VD) are the most common. AD is a representative cause of dementia, and once it occurs, there is no known treatment to return it to the pre-morbid state, and the gradual and irreversible decline in cognitive function adversely affects the lives of not only patients but also their caregivers, incurring significant economic and social burdens in our society (2). The clinical manifestations of dementia can generally be classified into three categories: (1) a significant decrease with respect to normative data in cognitive function, (2) the occurrence of peripheral symptoms of dementia, so-called behavioral and psychological symptoms of dementia (BPSD), and (3) the loss of autonomy in activities of daily living (3). BPSD, in particular, is not only related to poor prognosis in dementia patients, but it also increases the care burden for informal caregivers and worsens their quality of life (QoL) (4). In addition, the increased prevalence of BPSD and care burden are related to the worsening of caregivers' mental health (5). Therefore, the evaluation and management of BPSD is an important part of dementia management, along with strategies to delay cognitive decline in dementia patients.

A promising strategy for patients with dementia or patients at risk of AD is an individualized strategy (6, 7). Because BPSD is a combination of various symptoms, therapeutic strategies for patients with dementia can vary depending on each symptom (8). In addition, according to the current clinical evidence, BPSD may differ depending on the type of dementia (9). Therefore, identifying the type of dementia the patient has and the characteristics of BPSD associated with that type may be a promising strategy to effectively deal with BPSD and promote individualized management of dementia patients. However, there has not been any systematic review of the literature comparing the difference in the prevalence of BPSD by type of dementia in community-dwelling settings.

Therefore, the authors tried to synthesize the prevalence rates of BPSD characteristics in patients with dementia based on large-scale community-dwelling populations and determine the difference in the prevalence of each symptom that constitutes BPSD by dementia type. This study was limited to community-dwelling populations because referral and selection biases may exist in environments such as long-term care facilities (10).

## Materials and Methods

### Study Registration

The systematic review protocol was registered in the open science framework (OSF) registries (URL: https://osf.io/dmj7k). We reported this review according to the Preferred Reporting Items for Systematic Reviews and Meta-Analyses statement (Supplementary Material 1) (11).

### Data Sources and Search Strategy

One author (CYK) searched Medline *via* PubMed, EMBASE *via* Elsevier, and PsycARTICLES *via* ProQuest, to obtain relevant studies. The search date was December 5, 2020, and all studies published up to this search date were considered. In addition, a manual search was performed on the reference lists of eligible studies and relevant review articles to collect potentially missing literature. There were no language or publication status limitations. The authors were fluent in English, Chinese, Japanese, and Korean, and for papers written in a language other than those mentioned, Google translation or, if needed, a paid service request for translation was made to an academic translation company. The search strategy used for each database is presented in Supplementary Material 2.

### Inclusion Criteria

Regarding the study type, original clinical studies that enrolled at least 300 community-dwelling participants were included. The cutoff of 300 samples was based on the criteria of a recently published systematic review on the prevalence of dementia in Europe (12). In addition, more than 300 samples are generally considered to provide a reliable estimate of the effect size (13). For longitudinal studies, only baseline data were used. Regarding study populations, people with any type of dementia in community-dwelling settings were included. However, studies on dementia patients with other serious illnesses such as cancer and Down's syndrome, which can affect psychiatric symptoms of dementia patients and studies on patients with psychiatric disorders, which may mimic BPSD in dementia along with delirium, schizophrenia, bipolar disorder, major depressive disorder, post-traumatic stress disorder were excluded. There were no restrictions on the patient's current treatment status. Studies on dementia patients in nursing homes or hospitals, and studies that were unclear about targeting community-dwelling populations, studies of mixed samples (i.e., including samples other than community-dwelling dementia patients), and studies with unclear sample types and sizes were also excluded. Regarding outcomes, studies that used standardized diagnostic criteria or validated assessment tools for BPSD and studies reporting raw prevalence data on BPSD in community-dwelling dementia patients were included. However, studies that estimated the prevalence of BPSD by the rate of psychotropic drug use and studies that reported only symptom score or prevalence rate without raw prevalence data of BPSD in community-dwelling dementia patients were excluded.

### Study Selection

First, the authors (CYK and BL) independently screened the titles and abstracts of all searched articles to find potentially eligible studies. Second, full-texts of potentially eligible studies were retrieved independently by CYK and BL to determine whether those texts meet the inclusion criteria above. Disagreements were resolved through discussion.

### Data Extraction

A standardized pilot-tested form was used to extract data from the included studies to assess study quality and evidence synthesis. The extracted information included the first author's name, publication year, country, sample size, dropout rate, dementia type, dementia severity, mean age, disease duration in participants, assessment methods, and raw prevalence data of BPSD. However, for longitudinal studies, the baseline data were collected and analyzed. The authors (CYK and BL) extracted the data independently, and any discrepancies were resolved through discussion. Additional information was requested, the corresponding author was contacted about the included studies *via* e-mail if the data were insufficient or ambiguous.

### Quality Assessment

The methodological qualities of prevalence studies were assessed using the Joanna Briggs Institute's critical appraisal checklist (14). This tool assesses the quality of studies reporting prevalence data by assessing the following nine questions: *Was the sample frame appropriate to address the target population? Were study participants sampled appropriately? Was the sample size adequate? Were the study subjects and the setting described in detail? Was the data analysis conducted with sufficient coverage of the identified sample? Were valid methods used for the identification of the condition? Was the condition measured in a standard, reliable way for all participants? Was there appropriate statistical analysis?* Finally, *was the response rate adequate, and if not, was the low response rate managed appropriately?* (14). The authors (CYK and BL) independently assessed the methodological quality of the included studies, and any disagreement was resolved through discussion.

### Data Synthesis and Analysis

The prevalence of BPSD characteristics in community-dwelling dementia patients according to dementia type was analyzed. The Neuropsychiatric Inventory (NPI) (15), the most widely used tool for evaluating BPSD, was prioritized. However, other BPSD evaluation tools were also used. The main characteristics of all included studies were descriptively summarized. Regarding meta-analysis, STATA/MP software version 16 (StataCorp LLC, TX, USA) was used with the random-effects model. Specifically, the Metaprop command was used to estimate the prevalence of BPSD characteristics (16). The estimated prevalence of each BPSD symptom and its 95% confidence interval (CI) were calculated by meta-analysis. The overall prevalence rate was reprocessed with the Excel office 365 program (Microsoft, Redmond, WA). The I-squared statistic was used to evaluate the degree of heterogeneity of the studies, and I-squared values >50 and 75% indicated substantial and high heterogeneity, respectively. Using available data, the authors conducted subgroup analyses according to (a) type of dementia, (b) severity of dementia, and (c) mean age of participants. Mild dementia was considered if the Clinical Dementia Rating Scale (CDR) was 0.5 or 1, or the Mini-Mental State Examination (MMSE) score was between 21 and 25. Moderate dementia was considered if CDR was 2 or MMSE was between 11 and 20. Severe dementia was considered if CDR was 3 or more or MMSE was between 0 and 10. Participants up to 75 years of mean age were considered early elderly, and those over 76 years old were considered late elderly. Moreover, sensitivity analysis removing data outliers was performed to investigate the robustness of the meta-analysis results.

### Reporting Bias

For each meta-analysis, funnel plot was generated to evaluate the evidence of publication bias. However, it was meaningfully interpreted only when sufficient studies (more than 10 studies in each meta-analysis) were included.

## Results

### Description of Studies

**Figure 1:**
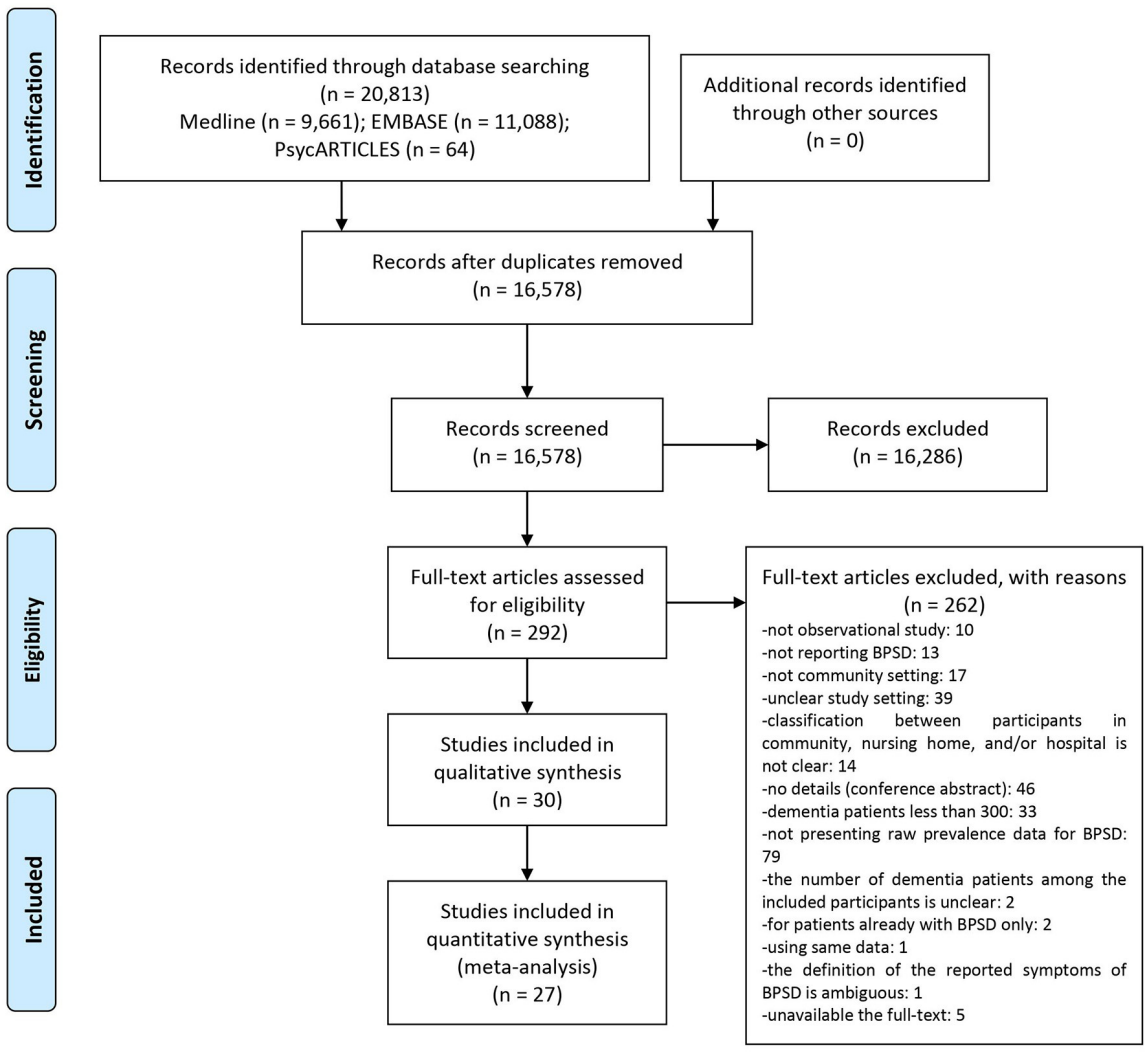
PRISMA flow chart of this review.

Among the searched 20,813 documents, titles and abstracts of 16,578 studies were screened after excluding duplicate documents. The initial screening yielded a review of the full-texts of 292 potentially eligible studies, of which 262 studies that did not meet the inclusion criteria were excluded (Supplementary Material 3). Finally, 30 studies were included in this review (17–46). Most studies were written in English, except for two in French (23, 24) and one in Chinese (25). Among them, 27 studies (17–33, 35–43, 46) were included in the meta-analysis (Figure 1).

### Characteristics of the Studies

Of the 30 studies included, 18 (17–21, 23–25, 28, 30, 34, 37–42, 44) were cross-sectional studies, and of the remaining studies, 10 (22, 26, 27, 29, 32, 33, 35, 36, 43, 46) were longitudinal studies, one (31) was a retrospective study, and one (45) was baseline data from two clinical trials. Twenty studies (18–22, 27, 31–33, 36, 39, 45) were conducted in the United States, six (23, 24, 26, 29, 30, 35) in France, two (25, 37) in China, and one in Sweden (17), Canada (28), Italy (34), Norway (40), Singapore (42), and Australia (43), respectively. The remaining four studies were conducted in several countries: one (41) in Latin America, China, and India, one (38) in Peru, Mexico, Venezuela, Puerto Rico, Cuba, India, China, and the Dominican Republic, and the other two (44) in European countries (including England, Estonia, Finland, France, Germany, Netherlands, Spain, and Sweden). Nineteen studies (17, 21, 23, 24, 26, 28–30, 32–35, 37, 38, 40, 41, 43, 44, 46) did not report the ethnicity of the participants. Otherwise, except for one study (25) involving only Chinese participants, the rest (18–20, 22, 27, 31, 36, 39, 42, 45) were multi-ethnic studies. The sample size (only for community-dwelling people with dementia) ranged from 324 to 3,768. Participants' type of dementia was not specified in 8 studies (17, 25, 28, 37, 40–42, 45), and they were considered mixed samples. Ten studies (18, 21, 22, 31–33, 38, 39, 44, 46) described that they recruited two or more types of dementia patients such as AD, VD, dementia with Lewy bodies (DLB), frontotemporal dementia (FTD), and/or other types. The remaining 12 studies (19, 20, 23, 24, 26, 27, 29, 30, 34–36, 43) were conducted among AD patients only. Participants' dementia diagnosis was determined using the Diagnostic and Statistical Manual of Mental Disorders (DSM) and/or the National Institute of Neurological Communicative Diseases and Stroke and the Alzheimer's Disease and Related Disorders Association (NINCDS/ADRDA) criteria in most studies (17–22, 25–30, 32–39, 41, 43). Seventeen studies described baseline dementia severity of participants by using MMSE score (17–20, 24, 26–30, 33–37, 43, 46). Except for six studies (23, 31, 32, 38, 40, 41) that did not report participants' age and five studies (18, 19, 28, 34, 36) targeting early elderly; all the remaining studies targeted late elderly participants. Disease duration was reported in 10 studies (24, 26, 30, 33–36, 43), from 8.92 months to 4.5 years. The most used assessment tool for BPSD evaluation was NPI, which was used in 21 studies (21–26, 28–30, 32–35, 37, 39–44, 46) (Table 1).

**Table 1:** Characteristics of the included studies.

**Study**	**Setting**	**Country**	**Ethnicity**	**Sample size (M:F)**	**Dementia type**	**Dementia diagnosis**	**Dementia severity**	**Mean age (yr)**	**Disease duration**	**Assessment of BPSD**	**Assessed by**
Forsell and Winblad (17)	CS	Sweden	NR	1,101 including 306 dementia patients (46:260)	NR	DSM-III-R	MMSE: -Without depression: 14.6 -With depression: 12.2	Without depression: 86.5 With depression: 87.6	NR	1. DSM-IV (major depression) 2. Comprehensive Psychopathological Rating Scale	Physicians
Klein et al. (18)	CS	US	Multiethnicity (White, etc.)	638 (212:426)	AD: 345 VD: 99 Other: 194	DSM-IV	MMSE: 16.6 ± 7.2	75.2 ± 10.2	4.0 ± 3.0 yr	1. Wandering behavior in the past 2 weeks 2. DSM-IV glossary definitions. (delusions, hallucinations) 3. DSM-IV criteria (sleep disorders) 4. CSDD (depression)	Clinicians by interviewing the patient and caregiver
Teri et al. (19)	CS	US	Multiethnicity (Caucasian, etc.)	523 (raw data was not presented for sex ratio)	AD	NINCDS-ADRDA criteria	MMSE: 20.4 ± 4.9	73.6 ± 6.9	NR	1.21-item behavior checklist (anxiety, depression, other problematic behaviors: present or absent)	Physician by observation of patient and informant
Bassiony et al. (20)	CS	US	Multiethnicity (Caucasian, African-American, Hispanic)	342 (82:260)	AD	NINCDS/ ADRDA criteria	MMSE: 14.7 ± 7.1	77.2 ± 9.1	4.5 ± 3.0 yr	1. DSM-IV (psychotic symptoms in the past 2 weeks)	Experienced geriatric psychiatrists using input from family members, caregivers and primary care physicians
Lyketsos et al. (21)	CS	US	NR	1,002 including 329 dementia patients (123:206)	AD: 214 VD: 62 Other: 53	DSM-IV	CDR 0.5, 1.0, 2, 3–5	84.2 ± 7.0	NR	1. NPI	Trained psychometrician or nurse
Lyketsos et al. (22)	LS	US	Multiethnicity (White and Black)	842 including 362 dementia patients (132:230)	AD: 258 VD: 86 PD: 6 Other: 12	DSM-IV, NINCDS/ ADRDA criteria	NR	77 ± 5.0	NR	1. NPI	Researchers by fully structured interview of informant
Arbus et al. (23)	CS	France	NR	578	AD	NR	Reisberg classification 2 or 3, 4, 5 or 6	NR	NR	1. NPI	NR
Rolland et al. (24)	CS	France	NR	571	AD	NR	MMSE: 10-29	Without wandering: 77.4 ± 7.1 With wandering: 77.6 ± 6.7	Without wandering: 1.1 ± 1.2 yr With wandering: 1.2 ± 1.2 yr	1. NPI	Based on a structured interview with a caregiver
Xie et al. (25)	CS	China	Chinese	1,540 including 373 dementia patients (223:150)	NR	DSM-IV	CDR 1 or 2	79.9 ± 9.8	NR	1. NPI	NR
Benoit et al. (26)	LS	France	NR	482 (140:342)	AD	DSM-IV, NINCDS/ ADRDA criteria	MMSE -Mild group: 23.5 ± 1.7 -Moderate group: 16.9 ± 2.6	Mild group: 77.2 ± 6.0 Moderate group:77.3 ± 7.8	Mild group: 38.9 ± 26.9 mon Moderate group: 47.4 ± 32.6 mon	1. NPI	Based on a structured interview with a caregiver
Wilson et al. (27)	LS	US	Multiethnicity (African American, etc.)	407 (134:273)	AD	NINCDS/ ADRDA criteria	MMSE -With hallucinations: 17.4 ± 4.1 -Without hallucinations: 19.7 ± 4.3	With hallucinations: 76.7 ± 6.6 Without hallucinations: 74.5 ± 7.7	NR	1. Questionnaire (hallucinations, delusions)	A structured interview administered by a trained research assistant
Peters et al. (28)	CS	Canada	NR	804 including 576 dementia patients (252:324)	NR	DSM-III	MMSE: 20.7 ± 5.6	73.0 ± 8.5	NR	1. NPI	NR
Rolland et al. (29)	LS	France	NR	682	AD	NINCDS/ ADRDA criteria	MMSE: 20.1 ± 4.5	77.4 ± 7	NR	1. NPI	NR
Benoit et al. (30)	CS	France	NR	686	AD	ICD-10, NINCDS/ ADRDA	MMSE: 20.0 ± 4.23 CDR 0.5, 1, 2, 3	77.9 ± 6.8	No apathy, no depression: 13.42 ± 13.09 mon Only depression: 8.92 ± 9.41 mon Only apathy: 13.51 ± 14.06 mon Both apathy and depression: 14.09 ± 14.82 mon	1. NPI	NR
Orengo et al. (31)	RS	US	Multiethnicity (African American, Hispanic, Caucasian, etc.)	385 (383:2)	AD: 82 VD: 70 Other: 226	ICD-9-CM	NR	NR	NR	1. RAS	Administered during a telephone screen (research staff)
Steinberg et al. (32)	LS	US	NR	408	AD: 255 VD: 44 Mixed: 27 Other: 82	Diagnosed in CCSMHA (DSM–III–R, NINCDS/ ADRDA)	CDR 0.5–1, 2, 3–5	NR	NR	1. NPI	Trained examiner, through a structured interview with the caregiver
Rao et al. (33)	LS	US	NR	449 (166:283)	AD: 271 AD + VD: 31 AD + other: 16 VD: 50 Other: 81	DSM-III-R NINCDS-ADRDA, NINDS-AIREN	MMSE -With TBI: 21.8 ± 5.9 -Without TBI: 21.3 ± 5.5	With TBI: 84.54 ± 5.3 Without TBI: 85.1 ± 6.5	MMSE -With TBI: 1.8 ± 1.4 yr -Without TBI: 1.9 ± 1.3 yr	1. NPI	Administered to caregivers or to persons very familiar with the participants
Spalletta et al. (34)	CS	Italy	NR	1,015 (292:723)	AD	NINCDS/ ADRDA	MMSE: 2.7 ± 0.1	74.6 ± 0.2	2.7 ± 0.1 yr	1. NPI (10-item)	An informant rated.
Arbus et al. (35)	LS	France	NR	686 (198:488)	AD	DSM-IV, NINCDS/ ADRDA	MMSE -No depression nor antidepressants at baseline: 20.43 ± 4.09 -Depression and/ or antidepressants at baseline: 19.65 ± 4.33	No depression nor antidepressants at baseline: 77.71 ± 7.03 Depression and/ or antidepressants at baseline: 77.99 ± 6.67	No depression nor antidepressants at baseline: 13.62 ± 13.40 mon Depression and/ or antidepressants at baseline: 12.73 ± 13.38 mon	1. NPI	Are read to the caregiver
Rountree et al. (36)	LS	US	Multiethnicity (White, etc.)	641 (205:436)	AD	NINCDS/ ADRDA	MMSE: 19.5 ± 6.64	73.0 ± 8.50	3.7 ± 2.29 yr	1. Questionnaire (hallucinations, delusions)	NR
Haibo et al. (37)	CS	China	NR	1,271	NR	DSM-IV	MMSE: 16.5 ± 6.3	80.9 ± 6.3	NR	1. NPI	Neuropsychiatrist
Andreasen et al. (38)	CS	Peru, Mexico, Venezuela, Puerto Rico, Cuba, India, China and Dominican Republic	NR	17,031 including 1,612 dementia patients (359:1,251)	AD: 424 VD: 244 DLB: 55 Unspecified: 889	DSM-IV 10/66 algorithm	NR	NR	NR	1. GMS-AGECAT	Employed researcher
Sadak et al. (39)	CS	US	Multiethnicity (White, Black, Hispanic, etc.)	3,768 (1,647:2,121)	AD: 3,338 DLB: 241 Behavioral variant FTD: 189	NINCDS-ADRDA (AD), Third report of the DLB Consortium (DLB), Consensus (behavioral variant FTD), NINDS-AIREN (VD)	CDR 1, 2, 3+	79 ± 6.98	NR	1. NPI-Q	Patient and family caregiver self-reports, review of medical records, and clinical evaluations
Wergeland et al. (40)	CS	Norway	NR	1,000 including 415 dementia patients (273:142)	NR	ICD-10	NR	NR	NR	1. NPI (10-item)	Physicians
Mograbi et al. (41)	CS	Latin America, China, India	NR	829	NR	DSM-IV, 10/ 66 criteria	CDR 0.5, 1, 2/ 3	NR	NR	1. NPI 2. ICD (depression)	Interview made with an informant
Vaingankar et al. (42)	CS	Singapore	Multiethnicity (Chinese, Malay, Indian)	399 (124:275)	Dementia	Semi-structured GMS-AGECAT along with CSI-D, CERAD test battery, and HAS-DDS, and also by applying the 10/66 protocol's diagnostic criteria	CDR: 2.2 ± 0.6	80.2 ± 0.3	NR	1. NPI	Trained lay interviewers to the older adults' informant
Connors et al. (43)	LS	Australia	NR	445 (222:223)	AD	DSM-IV	MMSE: 21.1 ± 5.3	78.7 ± 7.3	1.6 ± 1.9 yr	1. NPI (12-item)	A research nurse/ psychologist or specialist clinician
Costa et al. (44)	CS	European (England, Estonia, Finland, France, Germany, Netherlands, Spain, Sweden)	NR	1,997 including 1,217 community patients	AD, VD, Other (raw data was not presented)	Diagnosed by expert assessment (i.e, psychiatrist, neurologist, geriatrician, or general practitioner depending on countries' specific diagnostic procedures) and recorded in the medical record, MMSE score of 24 or below, and the presence of an informal caregiver (who visits at least twice a month)	NR	Agitation (+): 82.02 ± 0.22 Agitation (–): 82.88 ± 0.36	NR	1. NPI	As a structured interview with a knowledgeable informant
Lessing et al. (45)	Baseline data from two clinical trials	US	Multiethnicity (White, African-American, etc.)	509	Dementia	Physician diagnosis of dementia or MMSE scores of 23 or less	NR	82.6 ± 8.5	NR	1. ABID (aggression, agitation, rejection)	Caregiver-based rating
Holmstrand et al. (46)	LS	European (Finland, France, Germany, Netherlands, Spain, etc.)	NR	1,163 (431:732)	AD: 629 AD+VD: 71 VD: 186 FTD: 6 DLB: 23 Not specified: 185 Other: 54	NR	MMSE: median 15	With suicidal ideation: median 82 Without suicidal ideation: median 83	NR	1. NPI-Q 2. CSDD	Professionals in health or social care or by medical/ nursing/ social care students with practical experience and at least a Bachelor's degree

*ABID, Agitated Behavior in Dementia Scale; AD, Alzheimer's disease; BPSD, behavioral and psychological symptoms of dementia; CCSMHA, Cache County Study of Memory Health and Aging; CDR, clinical dementia rating; CERAD, Consortium to Establish a Registry for Alzheimer's Disease; CS, cross-sectional study; CSDD, Cornell Scale for Depression in Dementia; CSI-D, Community Screening Interview for Dementia; DLB, dementia with Lewy bodies; DSM, Diagnostic and Statistical Manual of Mental Disorders; FTD, frontotemporal dementia; GMS-AGECAT, Geriatric Mental State-Automated Geriatric Examination for Computer Assisted Taxonomy; HAS-DDS, History and Etiology Schedule–Dementia Diagnosis and Subtype; ICD, International Classification of Diseases; LS, longitudinal study; MMSE, Mini-Mental State Examination; NINCDS/ADRDA, National of Neurological Communicative Disease and Stroke and the Alzheimer's Disease and Related Disorders; NPI, neuropsychiatric inventory; NPI-Q, neuropsychiatric inventory-questionnaire; NR, nor recorded; PD, dementia due to Parkinson's disease; RAS, Ryden Aggression Scale; RS, retrospective study; US, United States; VD, vascular dementia*.

*CDR 0 = absent; 0.5 = questionable; 1 = present, but mild; 2 = moderate; 3 = severe; 4 = profound; and 5 = terminal*.

### Risk of Bias Assessment

Ten studies (18–20, 22, 27, 31, 36, 39, 42, 45), including multi-ethnic populations, were evaluated as “Yes” for the question “*Was the sample frame appropriate to address the target population?”* One study (25), including Chinese participants only, was evaluated as “No,” and remaining 19 studies (17, 21, 23, 24, 26, 28–30, 32–35, 37, 38, 40, 41, 43, 44, 46) without relevant information were evaluated as “Unclear.” In addition, 22 studies (17, 18, 20–22, 26–28, 30–36, 38–40, 42–44, 46) with consecutive sampling, random sampling or all permanent residents were evaluated as “Yes,” for the question “*Were study participants sampled appropriately?”* One (41) that excluded high-income earners and one (45) with two RCTs samples were evaluated as “No,” and six (19, 23–25, 29, 37) without a description of the sampling method were evaluated as “Unclear.” The sample size adequacy question (*Was the sample size adequate?*) showed that all studies did not present the calculation formula; however, we evaluated them as “Yes” because we included only studies with ≥ 300 sample size. For study subject description (*Were the study subjects and the setting described in detail?*), 22 studies (17, 19, 21, 23–25, 27–32, 34, 37–42, 44–46) were evaluated as “No” because they did not provide information, such as dementia duration, sex, race, type and severity of dementia, and the remaining eight (18, 20, 22, 26, 33, 35, 36, 43) were evaluated as “Yes” because they presented necessary information properly. All were evaluated as “Yes,” for data analysis coverage (*Was the data analysis conducted with sufficient coverage of the identified sample?)*, except for one study (44) that presented only “agitation cluster” prevalence without mentioning the symptom in detail. Except for three studies (19, 27, 36) that did not use valid symptom checklists or questionnaires, all other studies were evaluated as “Yes.” for the question “*Were valid methods used for the identification of the condition?”* Measurement reliability question (*Was the condition measured in a standard, reliable way for all participants?*) *showed that* except for six studies (23, 25, 28–30, 36) that did not specify the evaluator, all other studies were evaluated as, “Yes.” For the question, “*Was there appropriate statistical analysis?”* All studies were evaluated as “Yes.” Finally, for the question, “*Was the response rate adequate, and if not, was the low response rate managed appropriately?”* The studies reported responses from all the participants, and they were evaluated as “Not applicable” except for one study (41) that under-reported missing data (Table 2).

**Table 2:** Methodological quality of the included studies.

**Study**	***Was the sample frame appropriate to address the target population?***	***Were study participants sampled in an appropriate way?***	***Was the sample size adequate?***	***Were the study subjects and the setting described in detail?***	***Was the data analysis conducted with sufficient coverage of the identified sample?***	***Were valid methods used for the identification of the condition?***	***Was the condition measured in a standard, reliable way for all participants?***	***Was there appropriate statistical analysis?***	***Was the response rate adequate, and if not, was the low response rate managed appropriately?***
Forsell and Winblad (17)	Unclear	Yes	Yes	No	Yes	Yes	Yes	Yes	Not applicable
Klein et al. (18)	Yes	Yes	Yes	Yes	Yes	Yes	Yes	Yes	Not applicable
Teri et al. (19)	Yes	Unclear	Yes	No	Yes	Yes	Yes	Yes	Not applicable
Bassiony et al. (20)	Yes	Yes	Yes	Yes	Yes	Yes	Yes	Yes	Not applicable
Lyketsos et al. (21)	Unclear	Yes	Yes	No	Yes	Yes	Yes	Yes	Not applicable
Lyketsos et al. (22)	Yes	Yes	Yes	Yes	Yes	Yes	Yes	Yes	Not applicable
Arbus et al. (23)	Unclear	Unclear	Yes	No	Yes	Yes	Yes	Yes	Not applicable
Rolland et al. (24)	Unclear	Unclear	Yes	No	Yes	Yes	Yes	Yes	Not applicable
Xie et al. (25)	No	Unclear	Yes	No	Yes	Yes	Yes	Yes	Not applicable
Benoit et al. (26)	Unclear	Yes	Yes	Yes	Yes	Yes	Yes	Yes	Not applicable
Wilson et al. (27)	Yes	Yes	Yes	No	Yes	Yes	Yes	Yes	Not applicable
Peters et al. (28)	Unclear	Yes	Yes	No	Yes	Yes	Yes	Yes	Not applicable
Rolland et al. (29)	Unclear	Unclear	Yes	No	Yes	Yes	Yes	Yes	Not applicable
Benoit et al. (30)	Unclear	Yes	Yes	No	Yes	Yes	Yes	Yes	Not applicable
Orengo et al. (31)	Yes	Yes	Yes	No	Yes	Yes	Yes	Yes	Not applicable
Steinberg et al. (32)	Unclear	Yes	Yes	No	Yes	Yes	Yes	Yes	Not applicable
Rao et al. (33)	Unclear	Yes	Yes	Yes	Yes	Yes	Yes	Yes	Not applicable
Spalletta et al. (34)	Unclear	Yes	Yes	No	Yes	Yes	Yes	Yes	Not applicable
Arbus et al. (35)	Unclear	Yes	Yes	Yes	Yes	Yes	Yes	Yes	Not applicable
Rountree et al. (36)	Yes	Yes	Yes	Yes	Yes	Yes	No	Yes	Not applicable
Haibo et al. (37)	Unclear	Unclear	Yes	No	Yes	Yes	Yes	Yes	Not applicable
Andreasen et al. (38)	Unclear	Yes	Yes	No	Yes	Yes	Yes	Yes	Not applicable
Sadak et al. (39)	Yes	Yes	Yes	No	Yes	Yes	Yes	Yes	Not applicable
Wergeland et al. (40)	Unclear	Yes	Yes	No	Yes	Yes	Yes	Yes	Not applicable
Mograbi et al. (41)	Unclear	No	Yes	No	Yes	Yes	Yes	Yes	No
Vaingankar et al. (42)	Yes	Yes	Yes	No	Yes	Yes	Yes	Yes	Not applicable
Connors et al. (43)	Unclear	Yes	Yes	Yes	Yes	Yes	Yes	Yes	Not applicable
Costa et al. (44)	Unclear	Yes	Yes	No	No	Yes	Yes	Yes	Not applicable
Lessing et al. (45)	Yes	No	Yes	No	Yes	Yes	Yes	Yes	Not applicable
Holmstrand et al. (46)	Unclear	Yes	Yes	No	Yes	Unclear	Yes	Yes	Not applicable

### Prevalence of BPSD in Community-Dwelling Dementia Patients

A study by Lessing et al. (45), which was excluded from the quantitative synthesis, reported the prevalence of agitation, aggression, and rejection as 470/509 (92.34%), 323/509 (63.46%), and 277/509 (54.42%), respectively. Because our quantitative synthesis examined agitation and aggression together, and because the screening tools used in this study were heterogeneous, which is considered a potential cause of very high symptom prevalence, the prevalence in this study was not included in the quantitative synthesis. Also, a study by Costa et al. (44), which presented only prevalence of agitation cluster as 917/1,217 (75.35%) without mentioning the symptom in detail, was excluded from the quantitative synthesis. Finally, a study by Spalletta et al. (34) presented prevalence rates of psychotic, affective, and manic symptoms, respectively as 89/1,015 (8.77%), 297/1,015 (29.26%), and 51/1,015 (5.02%), was excluded from the quantitative synthesis. This is because the classification of BPSD used in this study was very heterogeneous compared to most other studies, making meta-analysis impossible.

**Figure 2:**
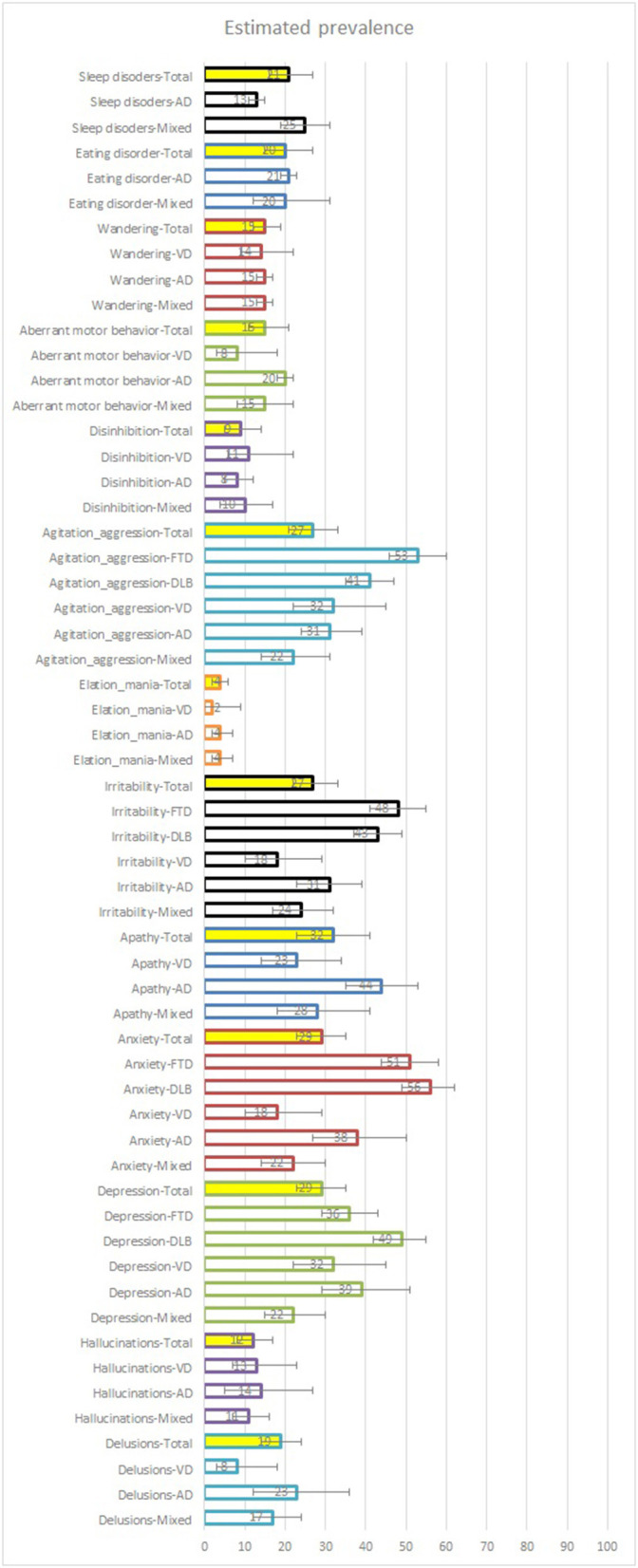
Estimated prevalence of BPSD symptoms. Mixed samples are sample data obtained from studies that did not specify the type of dementia that the participants had. AD, Alzheimer's disease; VD, vascular dementia.

Quantitative synthesis was performed for the prevalence of 13 BPSD characteristics. Regardless of the dementia type, the pooled estimated prevalence of the BPSD symptoms were as follows (in descending order): apathy was (32%, 95% CI: 23–41%), depression (29%, 95% CI: 23–35%), anxiety (29%, 95% CI: 23–35%), irritability (27%, 95% CI: 22–33%), agitation and aggression (27%, 95% CI: 21–33%). Sleep disorders (21%, 95% CI: 16–27%), eating disorder (20%, 95% CI: 15–27%), delusions (19%, 95% CI: 14–24%), aberrant motor behavior (15%, 95% CI: 11–21%), wandering (15%, 95% CI: 12–19%), hallucinations (12%, 95% CI: 8–17%), disinhibition (9%, 95% CI: 5–14%), and elation and mania (4%, 95% CI: 2–6%) (Figure 2; Table 3; Supplementary Material 4).

**Table 3:** Prevalence of BPSD in community-dwelling dementia patients.

**BPSD symptoms**	**Dementia type**	**Studies**	**Estimated prevalence (mean, 95% CI)**	***I*^**2−**^value**
Delusions	Mixed	12	17% (12–24%)	97.63%
	AD	7	23% (12–36%)	98.64%
	VD	1	8% (3–18%)	NA
	**Total**	**18**	**19% (14–24%)**	**97.96%**
Hallucinations	Mixed	12	11% (7–16%)	97.13%
	AD	8	14% (5–27%)	98.95%
	VD	1	13% (7–23%)	NA
	**Total**	**19**	**12% (8 to 17%)**	**98.10%**
Depression	Mixed	15	22% (15–30%)	60.39%
	AD	7	39% (29–51%)	98.53%
	VD	1	32% (22–45%)	NA
	DLB	1	49% (42–55%)	NA
	FTD	1	36% (29–43%)	NA
	**Total**	**20**	**29% (23–35%)**	**98.84%**
Anxiety	Mixed	12	22% (14–30%)	98.84%
	AD	6	38% (27–50%)	98.56%
	VD	1	18% (10–29%)	NA
	DLB	1	56% (49–62%)	NA
	FTD	1	51% (44–58%)	NA
	**Total**	**16**	**29% (23–35%)**	**98.73%**
Apathy	Mixed	11	28% (18–41%)	99.08%
	AD	4	44% (35–53%)	93.97%
	VD	1	23% (14–34%)	NA
	**Total**	**14**	**32% (23–41%)**	**98.84%**
Irritability	Mixed	11	24% (17–32%)	98.55%
	AD	5	31% (23–39%)	96.91%
	VD	1	18% (10–29%)	NA
	DLB	1	43% (37–49%)	NA
	FTD	1	48% (41–55%)	NA
	**Total**	**14**	**27% (22–33%)**	**97.97%**
Elation and mania	Mixed	10	4% (2–7%)	95.04%
	AD	4	4% (2–7%)	90.81%
	VD	1	2% (0–9%)	NA
	**Total**	**13**	**4% (2–6%)**	**93.59%**
Agitation and aggression	Mixed	13	22% (14–31%)	99.05%
	AD	5	31% (24–39%)	96.38%
	VD	1	32% (22–45%)	NA
	DLB	1	41% (35–47%)	NA
	FTD	1	53% (46–60%)	NA
	**Total**	**16**	**27% (21–33%)**	**98.67%**
Disinhibition	Mixed	11	10% (4–17%)	98.72%
	AD	4	8% (5–12%)	89.26%
	VD	1	11% (6–22%)	NA
	**Total**	**14**	**9% (5–14%)**	**98.18%**
Aberrant motor behavior	Mixed	10	15% (8–22%)	98.33%
	AD	4	20% (18–22%)	44.00%
	VD	1	8% (3–18%)	NA
	**Total**	**13**	**15% (11–21%)**	**97.48%**
Wandering	Mixed	2	15% (13–17%)	NA
	AD	2	15% (13–17%)	NA
	VD	1	14% (9–22%)	NA
	**Total**	**3**	**15% (12–19%)**	**79.85%**
Eating disorder	Mixed	6	20% (12–31%)	98.24%
	AD	3	21% (19–23%)	NA
	**Total**	**9**	**20% (15–27%)**	**97.21%**
Sleep disorders	Mixed	7	25% (19–31%)	96.06%
	VD	3	13% (11–15%)	NA
	**Total**	**10**	**21% (16–27%)**	**97.12%**

*AD, Alzheimer's disease; BPSD, behavioral and psychological symptoms of dementia; CI, confidence interval; DLB, dementia with Lewy bodies; FTD, frontotemporal dementia; NA, not applicable; VD, vascular dementia*.

*Mixed samples are sample data obtained from studies that did not specify the type of dementia that the participants had or studies that allowed more than one type of dementia*.

### Subgroup Analysis of the Prevalence of BPSD

#### Subgroup Analysis by Dementia Type

When analyzed according to the type of dementia, the pooled prevalence rates of BPSD characteristics in the mixed sample were as follows (in descending order): apathy (28%, 95% CI: 18–41%), sleep disorders (25%, 95% CI: 19–31%), irritability (24%, 95% CI: 17–32%), depression (22%, 95% CI: 15–30%), anxiety (22%, 95% CI: 14–30%), agitation and aggression (22%, 95% CI: 14–31%). Follwed by, eating disorder (20%, 95% CI: 12–31%), delusions (17%, 95% CI:12–24%), aberrant motor behavior (15%, 95% CI: 8–22%), wandering (15%, 95% CI: 13–17%), hallucinations (11%, 95% CI: 7–16%), disinhibition (10%, 95% CI: 4–17%), and elation and mania (4%, 95% CI: 2–7%). The pooled estimated prevalence of BPSD characteristics in AD patients were as follows (in descending order): apathy (44%, 95% CI: 35–53%), depression (39%, 95% CI: 29–51%), anxiety (38%, 95% CI: 27–50%), irritability (31%, 95% CI: 23–39%), agitation and aggression (31%, 95% CI: 24–39%), delusions (23%, 95% CI: 12–36%), eating disorder (21%, 95% CI: 19–23%). Followed by aberrant motor behavior (20%, 95% CI: 18–22%), wandering (15%, 95% CI: 13–17%), hallucinations (14%, 95% CI: 5–27%), sleep disorder (13%, 95% CI: 11–15%), disinhibition (8%, 95% CI: 5–12%), and elation and mania (4%, 95% CI: 2–7%). The pooled estimated prevalence of BPSD characteristics in VD patients were as follows (in descending order): depression (32%, 95% CI: 22–45%), agitation and aggression (32%, 95% CI: 22–45%), apathy (23%, 95% CI: 14–34%), anxiety (18%, 95% CI: 10–29%), irritability (18%, 95% CI: 10–29%). Followed by wandering (14%, 95% CI: 9–22%), hallucinations (13%, 95% CI: 7–23%), disinhibition (11%, 95% CI: 6–22%), delusions (8%, 95% CI: 3–18%), aberrant motor behavior (8%, 95% CI: 3–18%), and elation and mania (2%, 95% CI: 0–9%). The pooled estimated prevalence of BPSD characteristics in DLB patients were as follows (in descending order): anxiety (56%, 95% CI: 49–62%), depression (49%, 95% CI: 42–55%), irritability (43%, 95% CI: 37–49%), and agitation and aggression (41%, 95% CI: 35–47%). Finally, the pooled estimated prevalence of BPSD characteristics in FTD patients were as follows (in descending order): agitation and aggression (53%, 95% CI: 46–60%), anxiety (51%, 95% CI: 44–58%), irritability (48%, 95% CI: 41–55%), and depression (36%, 95% CI: 29–43%) (Figure 2; Table 4; and Supplementary Material 4).

**Table 4:** Subgroup analysis of estimated prevalence of BPSD symptoms (mean, 95% CI).

	**Total**	**Subgroup 1** **(dementia type)**				**Subgroup 2** **(dementia severity)**				**Subgroup 3** **(participant's age)**	
**BPSD**		**AD only**	**VD only**	**DLB only**	**FTD only**	**Mixed sample**	**Mild**	**Moderate**	**Severe**	**Early elderly**	**Late elderly**
Delusions	19% (14 to 24%)	23% (12–36%)	8% (3–18%)^*^	No data	No data	17% (12–24%)	14% (12–17%)^*^	23% (14–33%)	No data	35% (18–54%)^*^	16% (11–22%)
Hallucinations	12% (8–17%)	14% (5–27%)	13% (7–23%)^*^	No data	No data	11% (7–16%)	7% (5–9%)^*^	14% (7–23%)	No data	21% (6–42%)^*^	9% (5–14%)
Depression	29% (23–35%)	39% (29–51%)	32% (22–45%)^*^	49% (42–55%)^*^	36% (29–43%)^*^	22% (15–30%)	35% (28–41%)^*^	33% (22–45%)	34% (30–39%)^*^	42% (12–76%)^*^	26% (18–35%)
Anxiety	29% (23–35%)	38% (27–50%)	18% (10–29%)^*^	56% (49–62%)^*^	51% (44–58%)^*^	22% (14–30%)	30% (14–50%)^*^	36% (24–49%)	42% (37–46%)^*^	52% (49–55%)^*^	25% (17–34%)
Apathy	32% (23–41%)	44% (35–53%)	23% (14–34%)^*^	No data	No data	28% (18–41%)	28% (25–32%)^*^	44% (27–62%)	No data	59% (55–63%)^*^	33% (21–47%)
Irritability	27% (22–33%)	31% (23–39%)	18% (10–29%)^*^	43% (37–49%)^*^	48% (41–55%)^*^	24% (17–32%)	30% (18–44%)^*^	34% (26–43%)	39% (34–43%)^*^	43% (39–47%)^*^	26% (20–33%)
Elation and mania	4% (2–6%)	4% (2–7%)	2% (0–9%)^*^	No data	No data	4% (2–7%)	4% (2–7%)^*^	6% (4–9%)	No data	9% (6–11%)^*^	4% (3–7%)
Agitation and aggression	27% (21–33%)	31% (24–39%)	32% (22–45%)^*^	41% (35–47%)^*^	53% (46–60%)^*^	22% (14–31%)	26% (13–42%)^*^	32% (20–46%)	56% (51–61%)^*^	36% (32–40%)^*^	25% (16–34%)
Disinhibition	9% (5–14%)	8% (5–12%)	11% (6–22%)^*^	No data	No data	10% (4–17%)	7% (5–9%)^*^	12% (4–23%)	No data	27% (24–31%)^*^	8% (3–14%)
Aberrant motor behavior	15% (11–21%)	20% (18–22%)	8% (3–18%)^*^	No data	No data	15% (8–22%)	10% (7–12%)^*^	24% (16–32%)	No data	36% (32–40%)^*^	17% (11–23%)
Wandering	15% (12–19%)	15% (13–17%)^*^	14% (9–22%)^*^	No data	No data	15% (13–17%)^*^	No data	15% (13–17%)^*^	No data	17% (15–21%)^*^	12% (11–14%)^*^
Eating disorder	20% (15–27%)	21% (19–23%)^*^	No data	No data	No data	20% (12–31%)	22% (17–27%)^*^	22% (15–31%)	No data	32% (29–36%)^*^	19% (13–26%)
Sleep disorders	21% (16–27%)	13% (11–15%)^*^	No data	No data	No data	25% (19–31%)	17% (13–22%)^*^	21% (14–29%)	No data	26% (24–29%)^*^	17% (14–21%)

*AD, Alzheimer's disease; BPSD, behavioral and psychological symptoms of dementia; CI, confidence interval; DLB, dementia with Lewy bodies; FTD, frontotemporal dementia; NA, not applicable; VD, vascular dementia*.

* *Results synthesized in three or less studies. Mixed samples are sample data obtained from studies that did not specify the type of dementia that the participants had or studies that allowed more than one type of dementia*.

#### Subgroup Analysis by Dementia Severity

Among the studies that reported the participant's dementia severity at baseline, the dementia severity in the selected studies could classify the severity as mild, moderate, and severe. However, the pooled estimated prevalence of BPSD characteristics in dementia patients with severe symptoms was only available for depression, anxiety, irritability, and agitation and aggression. Among the four symptoms, mild, moderate, and severe estimated prevalence rates of anxiety (30%, 95% CI: 14–50%; 36%, 95% CI: 24–49%; 42%, 95% CI: 37–46%), irritability (30%, 95% CI: 18–44%; 34%, 95% CI: 26–43%; 39%, 95% CI: 34–43%), and agitation and aggression (26%, 95% CI: 13–42%; 32%, 95% CI: 20–46%; 56%, 95% CI: 51–61%), respectively, showed a tendency to increase with increasing dementia severity. However, that of depression (35%, 95% CI: 28–41%; 33%, 95% CI: 22–45%; 34%, 95% CI: 30–39%) maintained a relatively consistently high level in all severity levels. Among the BPSD investigated, the prevalence of depression (33–35%), anxiety (30–42%), apathy (30–42%), and irritability (30–39%) was high, whereas those of hallucinations (7–14%), elation and mania (4–6%), and disinhibition (7–12%) were low, regardless of the severity (Table 4; Supplementary Material 4).

#### Subgroup Analysis by Age

Based on age 75 years as the cutoff point, participants' mean age at baseline was classified into early elderly and late elderly; however, only four studies (18, 19, 28, 36) reported data of early elderly participants. The prevalence of all BPSD symptoms was higher in the early elderly than in the late elderly. Of which the respective prevalence in the early elderly was more than double that of the late elderly for delusions (35%, 95% CI: 18–54% vs. 16%, 95% CI: 11–22%), hallucinations (21%, 95% CI: 6–42% vs. 9%, 95% CI: 5–14%), and anxiety (52%, 95% CI: 49–55% vs. 25%, 95% CI: 17–34%). The same trend was observed for elation and mania (9%, 95% CI: 6–11% vs. 4%, 95% CI: 3–7%), disinhibition (27%, 95% CI: 24–31% vs. 8%, 95% CI: 3–14%), and aberrant motor behavior (36%, 95% CI: 32–40% vs. 17%, 95% CI: 11–23%). Among the BPSD investigated, the prevalence of depression (26–42%), anxiety (25–52%), apathy (33–59%), and irritability (26–43%) was high, whereas those of hallucinations (9–21%), elation and mania (4–9%), and disinhibition (8–27%) were low, regardless of the participant's age (Table 4; Supplementary Material 4).

### Sensitivity Analysis

For sensitivity analyses, data outliers were removed in several meta-analyses, including delusions, hallucinations, depression, anxiety, disinhibition, aberrant motor behavior, and sleep disorders. The frequent data outliers were Teri et al. (19), Wilson et al. (27), Peters et al. (28), Rountree et al. (36), and Holmstrand et al. (46). Thus, the sensitivity analyzes had no significant impact on the overall meta-analysis results (Supplementary Material 4).

### Publication Bias

Funnel plots were generated for all meta-analysis results. Overall, the reported data showed high heterogeneity. Moreover, visual symmetry was confirmed in the funnel plot for only a few cases, including hallucinations with subgroup analyses of dementia severity and age of participants (in sensitivity analysis), elation and mania with subgroup analyses of dementia type, dementia severity, age of participants, and aberrant motor behavior with subgroup analysis dementia type (in sensitivity analysis) (Supplementary Material 4).

## Discussion

### Summary of Findings

In this systematic review, the prevalence of BPSD in community-dwelling populations with dementia was analyzed from previously published 30 large-scale community-based studies (17–46). In particular, subgroup analyses according to the type of dementia, the severity of dementia, age of participants, as well as the individual prevalence of BPSD were conducted. Analyses of the prevalence of 13 BPSD characteristics were conducted. Overall, the prevalence of each BPSD characteristic ranged from 4 (elation and mania) to 32% (apathy) in the pooled samples, from 4 (elation and mania) to 28% (apathy) in the mixed samples, 4 (elation and mania) to 44% (apathy) in AD patients only. The same trend was observed from 2 (elation and mania) to 32% (depression) in VD patients only, 41 (agitation and aggression) to 56% (anxiety) in DLB patients only, and 36 (depression) to 53% (agitation and aggression) in FTD patients only. The prevalence rates of some BPSD characteristics were similar regardless of the type of dementia in the population. The prevalence rates of hallucinations, elation and mania, and disinhibition were low, and the rates were under 15% in the total sample as well as both AD and VD patients only. The prevalence rates of depression and agitation and aggression were high (nearly 30%), mostly in all the sample groups. Interestingly, the prevalence of some BPSD characteristics differed according to the type of dementia. In six of these cases, the prevalence of each symptom in AD patients was 1.72 to 2.88 times greater than that in VD patients: delusions (23% in AD vs. 8% in VD); anxiety (38 vs. 18%); apathy (44 vs. 23%); irritability (31 vs. 18%); elation and mania (4 vs. 2%); and aberrant motor behavior (20 vs. 8%). However, the prevalence of disinhibition in VD patients was 1.38 times greater than that in AD patients (8 vs. 11%). Little differences were found between AD and VD populations for the prevalence of the three symptoms, including hallucinations (14 vs. 13%), agitation and aggression (31 vs. 32%), and wandering (15 vs. 14%). There was not enough data to analyze eating disorders and sleep disorders. Prevalence data of BPSD in DLB and FTD populations were available only for depression, anxiety, irritability, agitation and aggression, and the overall prevalence was higher than 30%. However, since most of the prevalence data on patients with VD, DLB, and FTD are based on three or fewer studies, the reliability cannot be considered high.

Subgroup analysis according to the participant's dementia severity at baseline showed three patterns. (a) The prevalence of symptoms tended to increase as the severity increased: anxiety (30% in mild; 36% in moderate; 42% in severe), irritability (30; 34; 39%), agitation and aggression (26; 32; 56%), delusion (14; 23%; no data), hallucinations (7; 14%; no data), apathy (28; 44%; no data), disinhibition (7; 12%; no data), and aberrant motor behavior (10; 24%; no data). (b) The prevalence of symptom maintained a relatively consistent moderate-to-high level in all severity: depression (35; 33; 34%), eating disorder (22; 22%; no data), and sleep disorders (17; 21%; no data); and (c) the prevalence of symptoms maintained a relatively consistently low level in all severity: elation and mania (4; 6%; no data). As for wandering, there were only data on moderate severity (15%); therefore, it was difficult to evaluate the difference according to severity. Subgroup analysis according to the participants' age showed that the prevalence rates of all BPSD characteristics in early elderly were 1.42 (wandering: 17% in early elderly vs. 12% in late elderly) to 3.5 (disinhibition: 27 vs. 8%) times greater than those in late elderly.

In evaluating the methodological quality of the studies using the Joanna Briggs Institute's critical appraisal checklist (14), all studies included an appropriate number of participants, and BPSD was identified with appropriate assessment tools, and statistical analysis was appropriately performed in most studies. However, it was discovered that the sampling method and study subject and setting were not described in detail in several studies.

### Differences From Previous Studies

Some systematic reviews on the prevalence of BPSD have been conducted before this current review. However, the current systematic review presents findings that are different from the previous studies. For example, van der Linde et al. (47) included 59 studies and analyzed the longitudinal persistence and incidence of individual symptoms of BPSD. The study found that prevalence of depression (8–57%), anxiety (17–52%), apathy (19–51%), irritability (6–57%), and agitation (18–87%) were high in patients with dementia (47), which is consistent with our findings. Interestingly, this study also found that hyperactivity (i.e., irritability, agitation, and wandering) and apathy showed high persistence and incidence, whereas depression and anxiety showed low or moderate persistence and moderate incidence, and psychotic symptoms showed low persistence with moderate or low incidence (47). This study presents original and valuable findings but does not consider the type of dementia that may affect the occurrence of BPSD, and the authors stated that the heterogeneity of the included studies and environmental factors, which may affect BPSD, need to be considered (47). In this respect, the current findings have a distinctive strength in that, we attempt to consider the heterogeneity of the type of dementia, dementia severity, participant's age, and study settings. In particular, our study highlights that the prevalence rates of anxiety, apathy, and irritability were high in the dementia population but may be higher in patients with AD compared to patients with VD. On the other hand, those of depression and agitation and aggression were both high in AD and VD with no significant difference. As the severity of dementia increased, the prevalence rates of these symptoms also tended to increase, and they were more common in the early elderly than in the late elderly.

Similarly, Zhao et al. included 48 articles and analyzed the prevalence rate of each BPSD symptom in AD patients (48). They emphasized that among BPSD symptoms, the prevalence rates of apathy (49%), depression (42%), aggression (40%), anxiety (39%), and sleep disorder (39%) was the highest in patients with AD (48), which is generally consistent with our findings in AD samples, except for sleep disorder (13%). However, only three studies supported the prevalence of sleep disorders in our review, potentially explaining these differences. Likewise, this study was similar to the current review, except that it was limited to AD patients, was based on studies with sample sizes of 50 or more, and the study settings were not limited (48). However, as shown in the meta-regression results of this study, the study setting may be a factor explaining the difference in the heterogeneity of BPSD prevalence across included studies (48). On the other hand, the current review targets a large sample of more than 300 participants and attempts to reduce potential heterogeneity by confining the study setting to community studies.

Finally, Feast et al. analyzed BPSD, especially in terms of challenges for family carers, including 25 high-quality studies (49). However, this study was not intended to estimate the prevalence of BPSD but to analyze the characteristics of the challenging behavior of patients with dementia for caregivers in family care settings. In addition, this systematic review did not also consider the type of dementia, dementia severity, participant's age, and study settings of the original studies (49). Therefore, the findings in the current review can help expand knowledge about BPSD by combining these findings with those in the existing reviews.

### Clinical Implications

Understanding individual BPSD characteristic is important in establishing individualized management strategies for dementia patients in clinical settings. Our analysis found frequent and rare symptoms depending on the type of dementia. According to our findings, apathy, depression, anxiety, irritability, agitation and aggression were common individual BPSD symptoms in AD patients, and depression and agitation and aggression were common in VD patients. In both cases, elation and mania was rare. Data on the prevalence of BPSD in patients with DLB and FTD were very limited due to the lack of relevant studies. Interestingly, as the severity of dementia evaluated by MMSE and/or CDR increased, the prevalence rates of most BPSD characteristics, including anxiety, irritability, agitation and aggression, delusion, hallucinations, apathy, disinhibition, and aberrant motor behavior, increased. However, in the other subgroup analysis, all BPSD prevalence was higher in the early elderly group than in the late elderly group. Considering that the severity of dementia usually increases with age, these results may seem contradictory. We present some hypotheses potentially relevant to this issue. **First**, the most important factor is the lack of data on early elderly among the studies included. The prevalence of BPSD in this population was all based on three or fewer studies. Therefore, in our findings, the prevalence of BPSD in patients with early elderly dementia may have been exaggerated by a small number of studies. **Second**, data on the prevalence of BPSD in severe dementia in this review were mostly absent. However, previously published studies have pointed out that the complaints of some BPSD may be underestimated as the cognitive decline of dementia patients increases (50). That is, the prevalence of BPSD in patients with severe dementia might be underestimated, and our review did not include enough data to investigate it. However, it could potentially be related to the lower prevalence of BPSD in the late elderly population compared to the early elderly population. **Third**, considering that most included studies were on the late elderly, the increase in the prevalence of BPSD with increased dementia severity may be more relevant data for late elderly dementia patients. In addition, dementia severity and the age of participants might be related to the prevalence of BPSD as independent factors.

### Strengths and Limitations

This study systematically reviewed large-scale studies of community-based populations for the first time to understand the characteristics of BPSD incidence in dementia patients. In addition, subgroup analysis according to the type of dementia, dementia severity, and age of participants were used to analyze potential factors related to the prevalence of BPSD. However, the results of this study should be interpreted carefully considering the following limitations:

**First**, since the data extracted from the studies included in this review were cross-sectional, they did not show the longitudinal trajectory of BPSD. Although our subgroup analysis estimated the trajectory of BPSD symptoms according to MMSE and/or CDR of participants and, it was not for the same population; therefore, it cannot be called a longitudinal trajectory in a strict sense. However, our study may present some findings that are useful for reference in future longitudinal studies. For example, a future longitudinal study could investigate whether some symptoms, such as anxiety, irritability, agitation and aggression, delusion, hallucinations, apathy, disinhibition, and aberrant motor behavior, will increase with increased dementia severity. Will others (e.g., depression, eating disorder, sleep disorders, and elation and mania) remain high or low, and will there be changes according to the progression of dementia and aging of participants? **Second**, one of the major limitations of this review is the heterogeneity in the characteristics of the included patients. Although we limited the studies included in this review about patients with dementia in community-dwelling settings, we cannot rule out the possibility of other various factors, such as the patient's country of residence, race/ethnicity, sex/gender, and underlying diseases, influencing BPSD occurrence. For example, according to a study comparing the prevalence of BPSD in dementia patients in Korea and the UK, the British participants had higher BPSD symptoms except for aggression than the Korean participants (51). However, studies are still required to compare the prevalence or severity of BPSD symptoms according to the population characteristics. This not only enables individualized dementia strategies in the future but can also help to understand the pathogenesis of dementia, including genetic-environmental interactions (52). **Third**, the lack of relevant studies to be included is also a weakness of this review. In particular, there were not enough studies for subgroup analysis, and most of the data on mild and severe VD, DLB, FTD, dementia, and the early elderly were based on three or fewer studies. There were rare reports of dementia type-specific prevalence of BPSD, other than that of AD. This is probably due to the low prevalence of other types of dementia. However, it is still important to identify individual BPSD characteristics in other types of dementia. For example, recently, a study of clinical neuroscience at the University of Cambridge has shown that apathy is an early marker of FTD, and it predicts subsequent cognitive decline (53). Likewise, understanding the occurrence of some BPSD may facilitate early management, as well as personalized management of dementia. In addition to the type of dementia, the prevalence of BPSD according to the severity of dementia, especially in severe dementia, was insufficiently reported. In terms of age, the prevalence of BPSD was mostly focused on the “late elderly.” Moreover, there may be other factors to consider to understand the differences in BPSD prevalence. For example, although not a large study, Indian researchers found that patients with late-onset AD had significantly higher severity of delusions, agitation, anxiety, disinhibition, and nighttime behavioral disturbances than those with early-onset AD (54). Also, a study in Japan, though not community-based, examining the relationship between severity of dementia and BPSD, demonstrated that patients with DLB did not show a significant difference in the NPI total score according to CDR staging (55). However, as the CDR increased in the AD group, the NPI total score also increased (55). The difference in the prevalence of BPSD according to patient age or the severity of dementia and potentially the difference between the types of dementia could be further investigated in a community-dwelling sample. **Fourth**, since this study only included large-scale studies involving more than 300 samples, it was not possible to detect the source of potential publication biases such as small-study effects in this field. In addition, heterogeneity of data and potential publication bias were confirmed in most meta-analysis results in the funnel plots. **Fifth**, the scope of our review is the prevalence of BPSD, but not the severity of BPSD. Therefore, the findings cannot be used as reference to confirm the severity of BPSD in community-dwelling patients with dementia. However, the severity of BPSD as well as the prevalence of BPSD has an important influence on the care burden; thus, it is a subject of high research value. **Finally**, the heterogeneity of the assessment tools used to evaluate the prevalence of BPSD in the included studies is also a major limitation of this systematic review. For example, even with the same conditions, heterogeneity in the prevalence of sleep disorders diagnosed by the DSM criteria (18) and the prevalence of sleep disorders screened by NPI (22) is inevitable. Moreover, in the included studies using NPI, there were studies in which the cut-off score for presence of BPSD was 1 point (21, 32, 35, 39, 41, 42), 4 points (22, 23, 25, 34, 37, 40, 44), or not specified (24, 26, 28–30, 33, 43, 46). The low cut-off score on the NPI for frequency of BPSD, are of poor clinical significance and risk to blur evidence increasing prevalence unrealistically. Therefore, our findings should be interpreted with caution.

### Suggestions for Future Research

Based on the findings and limitations of this review, we would like to suggest areas to consider in future research in this field. **First**, current large-scale community-based studies on BPSD lack long-term longitudinal follow-up. Long-term longitudinal follow-up studies will deepen our understanding of the characteristics of BPSD, especially in terms of persistence and incidence of individual BPSD according to the type and severity of dementia and the age of participants. **Second**, the patient's country of residence, race/ethnicity, sex/gender, and underlying diseases, as well as the type of dementia and the study setting should be fully considered as potential factors that can affect the characteristics of BPSD. Ideally, it would be possible to build a multinational cooperative community to compare the characteristics of BPSD across countries based on a homogeneous research setting. **Third**, comparing the BPSD characteristics of non-community-dwelling samples to those of community-dwelling samples may also be a promising research topic, though this may not have been of interest in this study. As described in the introduction section, the current review excluded nursing home or hospital inpatient samples due to referral and selection biases. Criteria for referral of BPSD patients to these long-term care facilities may vary cross-sectionally and/or longitudinally across cultures, regions, countries, and eras. Therefore, considering the possibility that there will be differences in these criteria for referral, it is thought that it will be possible in future studies to comprehensively review and compare the prevalence of BPSD according to each study setting.

## Conclusions

This systematic review tried to analyze the prevalence of BPSD in community-dwelling samples from 13 previously published large-scale studies. Overall, the pooled prevalence of apathy, depression, anxiety, irritability, agitation and aggression, sleep disorders, and eating disorder was higher than 20%, while that of disinhibition and elation and mania was lower than 10%. Interestingly, the prevalence of some BPSD characteristics differed according to the type of dementia. The prevalence of delusions, anxiety, apathy, irritability, elation and mania, and aberrant motor behavior in AD patients was 1.72–2.88 times greater than that in VD samples, while the prevalence of disinhibition in VD patients was 1.38 times greater than that in AD patients. Moreover, the prevalence of some symptoms, including anxiety, irritability, agitation and aggression, delusion, hallucinations, apathy, disinhibition, and aberrant motor behavior, tended to increase as the severity of dementia increased, while that of depression, eating disorder, sleep disorders, and elation and mania tended to be stable. The methodological quality of the included studies is not the best, and high heterogeneity may affect the certainty of the findings. However, the results of this review can deepen our understanding of the prevalence of BPSD.

## Data Availability Statement

The original contributions presented in the study are included in the article/Supplementary Material, further inquiries can be directed to the corresponding author/s.

## Author Contributions

The study was conceptualized by C-YK. The study search, study screening, data extraction, and quality assessment were conducted, and the manuscript was drafted by C-YK and BL. Both authors have read and approved the final manuscript.

## Funding

This research was supported by a grant from the Korea Health Technology R&D Project through the Korea Health Industry Development Institute (KHIDI), funded by the Ministry of Health and Welfare, Republic of Korea (Grant Number: HF20C0207).

## Conflict of Interest

The authors declare that the research was conducted in the absence of any commercial or financial relationships that could be construed as a potential conflict of interest.

## Publisher's Note

All claims expressed in this article are solely those of the authors and do not necessarily represent those of their affiliated organizations, or those of the publisher, the editors and the reviewers. Any product that may be evaluated in this article, or claim that may be made by its manufacturer, is not guaranteed or endorsed by the publisher.

## Abbreviations

AD: Alzheimer's disease
BPSD: behavioral and psychological symptoms of dementia
CDR: Clinical Dementia Rating Scale
CI: confidence interval
DLB: dementia with Lewy bodies
DSM: Diagnostic and Statistical Manual of Mental Disorders
FTD: frontotemporal dementia
MMSE: Mini-Mental State Examination
NINCDS/ADRDA: National Institute of Neurological Communicative Diseases and Stroke and the Alzheimer's Disease and Related Disorders Association
NPI: Neuropsychiatric Inventory
OSF: open science framework
QoL: quality of life
VD: vascular dementia.

## References

[1] World Health Organization. Dementia Cases Set to Triple by 2050 but Still Largely Ignored. Geneva: WHO (2012).

[2] Dharmarajan TS Gunturu SG. Alzheimer's disease: a healthcare burden of epidemic proportion. Am Health Drug Benef. (2009) 2:39–47. 25126271 PMC4106591

[3] Grand JH Caspar S Macdonald SW. Clinical features and multidisciplinary approaches to dementia care. J Multidiscip Healthc. (2011) 4:125–47. 10.2147/JMDH.S17773 21655340 PMC3104685

[4] Cerejeira J Lagarto L Mukaetova-Ladinska EB. Behavioral and psychological symptoms of dementia. Front Neurol. (2012) 3:73. 10.3389/fneur.2012.00073 22586419 PMC3345875

[5] Arthur PB Gitlin LN Kairalla JA Mann WC. Relationship between the number of behavioral symptoms in dementia and caregiver distress: what is the tipping point? Int Psychogeriatr. (2018) 30:1099–107. 10.1017/S104161021700237X 29143722 PMC7103581

[6] Isaacson RS Hristov H Saif N Hackett K Hendrix S Melendez J . Individualized clinical management of patients at risk for Alzheimer's dementia. Alzheimer Dementia J Alzheimer Assoc. (2019) 15:1588–602. 10.1016/j.jalz.2019.08.198 31677936 PMC6925647

[7] Manthorpe J Samsi K. Person-centered dementia care: current perspectives. Clin Interv Aging. (2016) 11:1733–40. 10.2147/CIA.S104618 27932869 PMC5135058

[8] Kim SK Park M. Effectiveness of person-centered care on people with dementia: a systematic review and meta-analysis. Clin Interv Aging. (2017) 12:381–97. 10.2147/CIA.S117637 28255234 PMC5322939

[9] Kazui H Yoshiyama K Kanemoto H Suzuki Y Sato S Hashimoto M . Differences of behavioral and psychological symptoms of dementia in disease severity in four major dementias. PLoS ONE. (2016) 11:e0161092. 10.1371/journal.pone.0161092 27536962 PMC4990196

[10] Makimoto K Kang Y Kobayashi S Liao XY Panuthai S Sung HC . Prevalence of behavioural and psychological symptoms of dementia in cognitively impaired elderly residents of long-term care facilities in East Asia: a cross-sectional study. Psychogeriatrics. (2019) 19:171–80. 10.1111/psyg.12380 30394003

[11] Moher D Liberati A Tetzlaff J Altman DG. Preferred reporting items for systematic reviews and meta-analyses: the PRISMA statement. PLoS Med. (2009) 6:e1000097. 10.1371/journal.pmed.1000097 19621072 PMC2707599

[12] Bacigalupo I Mayer F Lacorte E Di Pucchio A Marzolini F Canevelli M . A systematic review and meta-analysis on the prevalence of dementia in Europe: estimates from the highest-quality studies adopting the DSM IV diagnostic criteria. J Alzheimer Dis. (2018) 66:1471–81. 10.3233/JAD-180416 30412486 PMC6294583

[13] Bujang MA Adnan TH. Requirements for minimum sample size for sensitivity and specificity analysis. J Clin Diagn Res. (2016) 10:Ye01–6. 10.7860/JCDR/2016/18129.8744 27891446 PMC5121784

[14] Munn Z Moola S Lisy K Riitano D Tufanaru C. Methodological guidance for systematic reviews of observational epidemiological studies reporting prevalence and cumulative incidence data. Int J Evid Based Healthc. (2015) 13:147–53. 10.1097/XEB.0000000000000054 26317388

[15] Cummings JL Mega M Gray K Rosenberg-Thompson S Carusi DA Gornbein J. The Neuropsychiatric Inventory: comprehensive assessment of psychopathology in dementia. Neurology. (1994) 44:2308–14. 10.1212/WNL.44.12.2308 7991117

[16] Nyaga VN Arbyn M Aerts M. Metaprop: a stata command to perform meta-analysis of binomial data. Arch Public Health. (2014) 72:39. 10.1186/2049-3258-72-39 25810908 PMC4373114

[17] Forsell Y Winblad B. Major depression in a population of demented and nondemented older people: prevalence and correlates. J Am Geriatr Soc. (1998) 46:27–30. 10.1111/j.1532-5415.1998.tb01009.x 9434662

[18] Klein DA Steinberg M Galik E Steele C Sheppard JM Warren A . Wandering behaviour in community-residing persons with dementia. Int J Geriatr Psychiatry. (1999) 14:272–9. 10.1002/(SICI)1099-1166(199904)14:4<272::AID-GPS896>3.0.CO;2-P 10340188

[19] Teri L Ferretti LE Gibbons LE Logsdon RG McCurry SM Kukull WA . Anxiety of Alzheimer's disease: prevalence, and comorbidity. J Gerontol A Biol Sci Med Sci. (1999) 54:M348–52. 10.1093/gerona/54.7.M348 10462166

[20] Bassiony MM Steinberg MS Warren A Rosenblatt A Baker AS Lyketsos CG. Delusions and hallucinations in Alzheimer's disease: prevalence and clinical correlates. Int J Geriatr Psychiatry. (2000) 15:99–107. 10.1002/(SICI)1099-1166(200002)15:2<99::AID-GPS82>3.0.CO;2-5 10679840

[21] Lyketsos CG Steinberg M Tschanz JT Norton MC Steffens DC Breitner JC. Mental and behavioral disturbances in dementia: findings from the Cache County Study on Memory in Aging. Am J Psychiatry. (2000) 157:708–14. 10.1176/appi.ajp.157.5.708 10784462

[22] Lyketsos CG Lopez O Jones B Fitzpatrick AL Breitner J DeKosky S. Prevalence of neuropsychiatric symptoms in dementia and mild cognitive impairment: results from the cardiovascular health study. Jama. (2002) 288:1475–83. 10.1001/jama.288.12.1475 12243634

[23] Arbus C Andrieu S Amouyal-Barkate K Nourhashémi F Schmitt L Vellas B. Depressive symptoms in Alzheimer's disease: findings from the REAL.FR study. Revue de Medecine Interne. (2003) 24(Suppl. 3):325s. 10.1016/S0248-8663(03)80691-4 14710452

[24] Rolland Y Gillette-Guyonnet S Nourhashémi F Andrieu S Cantet C Payoux P . Wandering and Alzheimer's disease: descriptive study. REAL.FR program on Alzheimer's disease and field of care. Revue Med Interne. (2003) 24(Suppl. 3):333s–8s. 10.1016/S0248-8663(03)80692-6 14710453

[25] Xie HG Wang LN Yu X Wang W Yang LJ Ma TX . Neuropsychiatric symptoms in dementia and elderly people in the community: results from the Beijing Dementia Cooperative Study. Zhonghua Liu Xing Bing Xue Za Zhi. (2004) 25:829–32. 10.1016/S0197-4580(04)81105-9 15631732

[26] Benoit M Robert PH Staccini P Brocker P Guerin O Lechowski L . One-year longitudinal evaluation of neuropsychiatric symptoms in Alzheimer's disease. REALFR Study J Nutr Health Aging. (2005) 9:95–9. 15791352

[27] Wilson RS Krueger KR Kamenetsky JM Tang Y Gilley DW Bennett DA . Hallucinations and mortality in Alzheimer disease. Am J Geriatr Psychiatry. (2005) 13:984–90. 10.1097/00019442-200511000-00009 16286442

[28] Peters KR Rockwood K Black SE Bouchard R Gauthier S Hogan D . Characterizing neuropsychiatric symptoms in subjects referred to dementia clinics. Neurology. (2006) 66:523–8. 10.1212/01.wnl.0000198255.84842.06 16505306

[29] Rolland Y Andrieu S Cantet C Morley JE Thomas D Nourhashemi F . Wandering behavior and Alzheimer disease. REALFR Prospect Alzheimer Dis Assoc Disord. (2007) 21:31–8. 10.1097/WAD.0b013e31802f243e 17334270

[30] Benoit M Andrieu S Lechowski L Gillette-Guyonnet S Robert PH Vellas B. Apathy and depression in Alzheimer's disease are associated with functional deficit and psychotropic prescription. Int J Geriatr Psychiatry. (2008) 23:409–14. 10.1002/gps.1895 17918770

[31] Orengo CA Khan J Kunik ME Snow AL Morgan R Steele A . Aggression in individuals newly diagnosed with dementia. Am J Alzheimer's Dis Other Dement. (2008) 23:227–32. 10.1177/1533317507313373 18258723 PMC10846265

[32] Steinberg M Shao H Zandi P Lyketsos CG Welsh-Bohmer KA Norton MC . Point and 5-year period prevalence of neuropsychiatric symptoms in dementia: the Cache County Study. Int J Geriatr Psychiatry. (2008) 23:170–7. 10.1002/gps.1858 17607801 PMC2932652

[33] Rao V Rosenberg P Miles QS Patadia D Treiber K Bertrand M . Neuropsychiatric symptoms in dementia patients with and without a history of traumatic brain injury. J Neuropsychiatry Clin Neurosci. (2010) 22:166–72. 10.1176/jnp.2010.22.2.166 20463110 PMC2928219

[34] Spalletta G Musicco M Padovani A Rozzini L Perri R Fadda L . Neuropsychiatric symptoms and syndromes in a large cohort of newly diagnosed, untreated patients with Alzheimer disease. Am J Geriatr Psychiatry. (2010) 18:1026–35. 10.1097/JGP.0b013e3181d6b68d 20808086

[35] ArbusC GardetteV CantetCE AndrieuS NourhashémiF SchmittL . Incidence and predictive factors of depressive symptoms in Alzheimer's disease: the REALFR study. J Nutr Health Aging. (2011) 15:609–17. 10.1007/s12603-011-0061-121968854

[36] Rountree SD Chan W Pavlik VN Darby EJ Doody RS. Factors that influence survival in a probable Alzheimer disease cohort. Alzheimer Res Ther. (2012) 4:16. 10.1186/alzrt119 22594761 PMC3506931

[37] Haibo X Shifu X Pin NT Chao C Guorong M Xuejue L . Prevalence and severity of behavioral and psychological symptoms of dementia (BPSD) in community dwelling Chinese: findings from the Shanghai three districts study. Aging Ment Health. (2013) 17:748–52. 10.1080/13607863.2013.781116 23548031

[38] Andreasen P Lönnroos E von Euler-Chelpin MC. Prevalence of depression among older adults with dementia living in low- and middle-income countries: a cross-sectional study. Eur J Public Health. (2014) 24:40–4. 10.1093/eurpub/ckt014 23417621

[39] Sadak TI Katon J Beck C Cochrane BB Borson S. Key neuropsychiatric symptoms in common dementias: prevalence and implications for caregivers, clinicians, and health systems. Res Gerontol Nurs. (2014) 7:44–52. 10.3928/19404921-20130918-01 24079749 PMC3909707

[40] Wergeland JN Selbæk G Høgset LD Söderhamn U Kirkevold Ø. Dementia, neuropsychiatric symptoms, and the use of psychotropic drugs among older people who receive domiciliary care: a cross-sectional study. Int Psychogeriatr. (2014) 26:383–91. 10.1017/S1041610213002032 24252377

[41] Mograbi DC Ferri CP Stewart R Sosa AL Brown RG Laks J . Neuropsychological and behavioral disturbance correlates of unawareness of memory impairment in dementia: a population-based study. J Geriatr Psychiatry Neurol. (2015) 28:3–11. 10.1177/0891988714541868 25009158

[42] Vaingankar JA Chong SA Abdin E Picco L Jeyagurunathan A Seow E . Behavioral and psychological symptoms of dementia: prevalence, symptom groups and their correlates in community-based older adults with dementia in Singapore. Int Psychogeriatr. (2017) 29:1363–76. 10.1017/S1041610217000564 28416031

[43] Connors MH Ames D Woodward M Brodaty H. Psychosis and clinical outcomes in Alzheimer disease: a longitudinal study. Am J Geriatr Psychiatry. (2018) 26:304–13. 10.1016/j.jagp.2017.10.011 29174998

[44] Costa N Wübker A De Mauléon A Zwakhalen SMG Challis D Leino-Kilpi H . Costs of care of agitation associated with dementia in 8 European countries: results from the Right Time Place Care Study. J Am Med Direct Assoc. (2018) 19:95.e1–.e10. 10.1016/j.jamda.2017.10.013 29275939

[45] Lessing S Deck R Choi SSW. Impact of three dementia-related behaviors on caregiver depression: the role of rejection of care, aggression, and agitation. BMC Health Serv Res. (2019) 34:966–73. 10.1002/gps.5097 30897238 PMC6579654

[46] Holmstrand C Rahm Hallberg I Saks K Leino-Kilpi H Renom Guiteras A Verbeek H . Associated factors of suicidal ideation among older persons with dementia living at home in eight European countries. Aging Mental Health. (2021) 25:1730–9. 10.1080/13607863.2020.1745143 32223443

[47] van der Linde RM Dening T Stephan BC Prina AM Evans E Brayne C. Longitudinal course of behavioural and psychological symptoms of dementia: systematic review. Br J Psychiatry. (2016) 209:366–77. 10.1192/bjp.bp.114.148403 27491532 PMC5100633

[48] Zhao QF Tan L Wang HF Jiang T Tan MS Tan L . The prevalence of neuropsychiatric symptoms in Alzheimer's disease: systematic review and meta-analysis. J Affect Disord. (2016) 190:264–71. 10.1016/j.jad.2015.09.069 26540080

[49] Feast A Orrell M Charlesworth G Melunsky N Poland F Moniz-Cook E. Behavioural and psychological symptoms in dementia and the challenges for family carers: systematic review. Br J Psychiatry. (2016) 208:429–34. 10.1192/bjp.bp.114.153684 26989095 PMC4853642

[50] Lindbo A Gustafsson M Isaksson U Sandman PO Lövheim H. Dysphoric symptoms in relation to other behavioral and psychological symptoms of dementia, among elderly in nursing homes. BMC Geriatr. (2017) 17:206. 10.1186/s12877-017-0603-4 28882104 PMC5590234

[51] Shah A Ellanchenny N Suh GH. A comparative study of behavioral and psychological signs and symptoms of dementia in patients with dementia referred to psychogeriatric services in Korea and the United Kingdom. Int Psychogeriatr. (2004) 16:219–36. 10.1017/S1041610204000341 15318766

[52] Shah A Dalvi M Thompson T. Behavioural and psychological signs and symptoms of dementia across cultures: current status and the future. Int J Geriatr Psychiatry. (2005) 20:1187–95. 10.1002/gps.1417 16315147

[53] Malpetti M Jones PS Tsvetanov KA Rittman T van Swieten JC Borroni B . Apathy in presymptomatic genetic frontotemporal dementia predicts cognitive decline and is driven by structural brain changes. Alzheimer Dement. (2021) 17:969–83. 10.1002/alz.12252 33316852 PMC8247340

[54] Mushtaq R Pinto C Tarfarosh SFA Hussain A Shoib S Shah T . A comparison of the behavioral and psychological symptoms of dementia (BPSD) in early-onset and late-onset Alzheimer's disease - a study from South East Asia (Kashmir, India). Cureus. (2016) 8:e625. 10.7759/cureus.625 27433404 PMC4934927

[55] Hashimoto M Yatabe Y Ishikawa T Fukuhara R Kaneda K Honda K . Relationship between dementia severity and behavioral and psychological symptoms of dementia in dementia with lewy bodies and Alzheimer's disease patients. Dement Geriatr Cogn Dis Extra. (2015) 5:244–52. 10.1159/000381800 26195980 PMC4483492

