# Supplementary material for: Prevalence of Behavioral and Psychological Symptoms of Dementia in Community-Dwelling Dementia Patients: A Systematic Review
Source: Front Psychiatry. 2021 Oct 21;12:741059. doi: 10.3389/fpsyt.2021.741059 (PMC8566725; doi:10.3389/fpsyt.2021.741059)
Supplement: Supplementary file 1 [file Data_Sheet_1.docx]

**Supplement material 1. PRISMA 2009 Checklist**

| **Section/topic** | **#** | **Checklist item** | **Reported on page #** |
| --- | --- | --- | --- |
| **TITLE** | | |  |
| Title | 1 | Identify the report as a systematic review, meta-analysis, or both. | 1 |
| **ABSTRACT** | | |  |
| Structured summary | 2 | Provide a structured summary including, as applicable: background; objectives; data sources; study eligibility criteria, participants, and interventions; study appraisal and synthesis methods; results; limitations; conclusions and implications of key findings; systematic review registration number. | 2 |
| **INTRODUCTION** | | |  |
| Rationale | 3 | Describe the rationale for the review in the context of what is already known. | 3 |
| Objectives | 4 | Provide an explicit statement of questions being addressed with reference to participants, interventions, comparisons, outcomes, and study design (PICOS). | 3 |
| **METHODS** | | |  |
| Protocol and registration | 5 | Indicate if a review protocol exists, if and where it can be accessed (e.g., Web address), and, if available, provide registration information including registration number. | 4 |
| Eligibility criteria | 6 | Specify study characteristics (e.g., PICOS, length of follow-up) and report characteristics (e.g., years considered, language, publication status) used as criteria for eligibility, giving rationale. | 4-5 |
| Information sources | 7 | Describe all information sources (e.g., databases with dates of coverage, contact with study authors to identify additional studies) in the search and date last searched. | 4 |
| Search | 8 | Present full electronic search strategy for at least one database, including any limits used, such that it could be repeated. | 4 |
| Study selection | 9 | State the process for selecting studies (i.e., screening, eligibility, included in systematic review, and, if applicable, included in the meta-analysis). | 5 |
| Data collection process | 10 | Describe method of data extraction from reports (e.g., piloted forms, independently, in duplicate) and any processes for obtaining and confirming data from investigators. | 5 |
| Data items | 11 | List and define all variables for which data were sought (e.g., PICOS, funding sources) and any assumptions and simplifications made. | 5 |
| Risk of bias in individual studies | 12 | Describe methods used for assessing risk of bias of individual studies (including specification of whether this was done at the study or outcome level), and how this information is to be used in any data synthesis. | 5 |
| Summary measures | 13 | State the principal summary measures (e.g., risk ratio, difference in means). | 5-6 |
| Synthesis of results | 14 | Describe the methods of handling data and combining results of studies, if done, including measures of consistency (e.g., I^2^) for each meta-analysis. | 6 |

| **Section/topic** | **#** | **Checklist item** | **Reported on page #** |
| --- | --- | --- | --- |
| Risk of bias across studies | 15 | Specify any assessment of risk of bias that may affect the cumulative evidence (e.g., publication bias, selective reporting within studies). | 7 |
| Additional analyses | 16 | Describe methods of additional analyses (e.g., sensitivity or subgroup analyses, meta-regression), if done, indicating which were pre-specified. | 6-7 |
| **RESULTS** | | |  |
| Study selection | 17 | Give numbers of studies screened, assessed for eligibility, and included in the review, with reasons for exclusions at each stage, ideally with a flow diagram. | 7 |
| Study characteristics | 18 | For each study, present characteristics for which data were extracted (e.g., study size, PICOS, follow-up period) and provide the citations. | 7 |
| Risk of bias within studies | 19 | Present data on risk of bias of each study and, if available, any outcome level assessment (see item 12). | 8 |
| Results of individual studies | 20 | For all outcomes considered (benefits or harms), present, for each study: (a) simple summary data for each intervention group (b) effect estimates and confidence intervals, ideally with a forest plot. | 8-10 |
| Synthesis of results | 21 | Present results of each meta-analysis done, including confidence intervals and measures of consistency. | 8-10 |
| Risk of bias across studies | 22 | Present results of any assessment of risk of bias across studies (see Item 15). | 11 |
| Additional analysis | 23 | Give results of additional analyses, if done (e.g., sensitivity or subgroup analyses, meta-regression [see Item 16]). | 9-10 |
| **DISCUSSION** | | |  |
| Summary of evidence | 24 | Summarize the main findings including the strength of evidence for each main outcome; consider their relevance to key groups (e.g., healthcare providers, users, and policy makers). | 12-13 |
| Limitations | 25 | Discuss limitations at study and outcome level (e.g., risk of bias), and at review-level (e.g., incomplete retrieval of identified research, reporting bias). | 14-16 |
| Conclusions | 26 | Provide a general interpretation of the results in the context of other evidence, and implications for future research. | 17 |
| **FUNDING** | | |  |
| Funding | 27 | Describe sources of funding for the systematic review and other support (e.g., supply of data); role of funders for the systematic review. | 17 |

*From:*  Moher D, Liberati A, Tetzlaff J, Altman DG, The PRISMA Group (2009). Preferred Reporting Items for Systematic Reviews and Meta-Analyses: The PRISMA Statement. PLoS Med 6(7): e1000097. doi:10.1371/journal.pmed1000097

**Supplement material 2. Search strategies used in each database**

**MEDLINE via PubMed**

|  | Searches | Results |
| --- | --- | --- |
| #1 | Dementia[MH] OR dement*[TIAB] OR Alzheimer*[TIAB] OR “Lewy body”[TIAB] OR Huntington*[TIAB] OR Parkinson*[TIAB] OR “Pick disease”[TIAB] | 368,307 |
| #2 | “Behavioral Symptoms”[MH] OR “Psychotic Disorders”[MH] OR Emotion[MH] OR BPSD[TIAB] OR behavioral[TIAB] OR psychological[TIAB] OR neuropsychiatric[TIAB] OR psychotic[TIAB] | 1,059,197 |
| #3 | “Cohort Studies”[MH] OR “Cross-sectional Studies”[MH] OR “Epidemiologic Studies”[MH] OR “cohort design”[TIAB] OR “cohort stud*”[TIAB] OR “cross-sectional analysis”[TIAB] OR “cross-sectional design”[TIAB] OR “cross-sectional stud*”[TIAB] OR “epidemiologic stud*”[TIAB] OR incidence[TIAB] OR “longitudinal design”[TIAB] OR “longitudinal stud*”[TIAB] OR “observational stud*”[TIAB] OR “population stud*”[TIAB] OR prevalence[TIAB] | 3,699,233 |
| #4 | #1 AND #2 AND #3 | **9,661** |

**EMBASE via Elsevier**

|  | Searches | Results |
| --- | --- | --- |
| #1 | 'dementia'/exp OR dement*:ab,ti OR Alzheimer*:ab,ti OR ‘Lewy body’:ab,ti OR Huntington*:ab,ti OR Parkinson:ab,ti OR ‘Pick disease’:ab,ti | 553,534 |
| #2 | 'emotion'/exp OR 'psychosis'/exp OR BPSD:ab,ti OR behavioral:ab,ti OR psychological:ab,ti OR neuropsychiatric:ab,ti OR psychotic:ab,ti | 1,441,612 |
| #3 | 'cross-sectional study'/exp OR 'cohort design':ab,ti OR 'cohort stud*':ab,ti OR 'cross-sectional analysis':ab,ti OR 'cross-sectional design':ab,ti OR 'cross-sectional stud*':ab,ti OR 'epidemiologic stud*':ab,ti OR incidence:ab,ti OR 'longitudinal design':ab,ti OR 'longitudinal stud*':ab,ti OR 'observational stud*':ab,ti OR 'population stud*':ab,ti OR prevalence:ab,ti | 2,711,823 |
| #4 | #1 AND #2 AND #3 | **11,088** |

**PsycARTICLES via ProQuest**

|  | Searches | Results |
| --- | --- | --- |
| #1 | SU(Dementia) OR TI(dement*) OR AB(dement*) OR TI(Alzheimer*) OR AB(Alzheimer*) OR TI(Lewy body) OR AB(Lewy body) OR TI(Huntington*) OR AB(Huntington*) OR TI(Parkinson) OR AB(Parkinson) OR TI(Pick disease) OR AB(Pick disease) | 1,603 |
| #2 | SU(Behavioral Symptoms) OR SU(Psychotic Disorders) OR SU(Emotion) OR TI(BPSD) OR AB(BPSD) OR TI(behavioral) OR AB(behavioral) OR TI(psychological) OR AB(psychological) OR TI(neuropsychiatric) OR AB(neuropsychiatric) OR TI(psychotic) OR AB(psychotic) | 51,528 |
| #3 | SU(Cohort Studies) OR SU(Cross-sectional Studies) OR SU(Epidemiologic Studies) OR TI(cohort design) OR AB(cohort design) OR TI(cohort stud*) OR AB(cohort stud*) OR TI(cross-sectional analysis) OR AB(cross-sectional analysis) OR TI(cross-sectional design) OR AB(cross-sectional design) OR TI(cross-sectional stud*) OR AB(cross-sectional stud*) OR TI(epidemiologic stud*) OR AB(epidemiologic stud*) OR TI(incidence) OR AB(incidence) OR TI(longitudinal design) OR AB(longitudinal design) OR TI(longitudinal stud*) OR AB(longitudinal stud*) OR TI(observational stud*) OR AB(observational stud*) OR TI(population stud*) OR AB(population stud*) OR TI(prevalence) OR AB(prevalence) | 16,611 |
| #4 | #1 AND #2 AND #3 | **64** |

**Supplement material 3. Excluded studies after full-text review in this review**

**1) not observational study: 10**

1. Beck BJ. Neuropsychiatric manifestations of diffuse Lewy body disease. *Journal of geriatric psychiatry and neurology.* 1995;8(3):189-196.

2. Auer SR, Monteiro IM, Reisberg B. Behavioral symptoms in dementia: community-based research. *International psychogeriatrics.* 1996;8 Suppl 3:363-366; discussion 381-362.

3. Balestrieri M. [Expressions of depression in Alzheimer's disease. The current scientific debate]. *Epidemiologia e psichiatria sociale.* 2000;9(2):126-139.

4. Schneider LS, Dagerman KS. Psychosis of Alzheimer's disease: clinical characteristics and history. *Journal of psychiatric research.* 2004;38(1):105-111.

5. Meguro K. Clinical features of mild cognitive impairment and dementia in a community: an update of the Osaki-Tajiri Project. *The Tohoku journal of experimental medicine.* 2008;215(2):125-131.

6. Ryu SH. Course of neuropsychiatric symptoms in alzheimer's disease. *International psychogeriatrics.* 2013;25:S40-S41.

7. Sakai K, Yamane Y, Yamamoto Y, Maeda K. [Depression in dementia with Lewy bodies]. *Seishin shinkeigaku zasshi = Psychiatria et neurologia Japonica.* 2013;115(11):1127-1134.

8. Solla P, Cannas A, Mulas CS, Marrosu F. Delusional jealousy in Parkinson's disease patients with and without dementia. *Neurological Sciences.* 2014;35(8):1307-1308.

9. Craig K, Yee C, Mills B, et al. Neuropsychiatric symptoms in Alzheimer's disease (AD): How sensitive, how prevalent? *Psychological services.* 2016;201:99-100.

10. Nagata T, Shinagawa S, Nakajima S, Mimura M, Shigeta M. Association between Neuropsychiatric Improvement and Neurocognitive Change in Alzheimer's Disease: Analysis of the CATIE-AD Study. *Journal of Alzheimer's disease : JAD.* 2018;66(1):139-148.

**2) not reporting BPSD: 13**

1. Gallicchio L, Siddiqi N, Langenberg P, Baumgarten M. Gender differences in burden and depression among informal caregivers of demented elders in the community. *International journal of geriatric psychiatry.* 2002;17(2):154-163.

2. Andrieu S, Balardy L, Gillette-Guyonnet S, et al. Informal caregiver's burden of Alzheimer's patients: Assessment and factors associated. *Revue de Medecine Interne.* 2003;24(SUPPL. 3):351s-359s.

3. Schulz R, Belle SH, Czaja SJ, McGinnis KA, Stevens A, Zhang S. Long-term care placement of dementia patients and caregiver health and well-being. *Jama.* 2004;292(8):961-967.

4. Sweet RA, Bennett DA, Graff-Radford NR, Mayeux R. Assessment and familial aggregation of psychosis in Alzheimer's disease from the National Institute on Aging Late Onset Alzheimer's Disease Family Study. *Brain : a journal of neurology.* 2010;133(Pt 4):1155-1162.

5. Shim YS, Roe CM, Buckles VD, Morris JC. Clinicopathologic study of Alzheimer's disease: Alzheimer mimics. *Journal of Alzheimer's disease : JAD.* 2013;35(4):799-811.

6. Mittal D, Li C, Viverito K, et al. Monitoring for metabolic side effects among outpatients with dementia receiving antipsychotics. *Psychiatric services (Washington, DC).* 2014;65(9):1147-1153.

7. Panegyres PK, Chen HY. Early-onset Alzheimer's disease: A global cross-sectional analysis. *European journal of neurology.* 2014;21(9):1149-1141e1165.

8. Liu S, Li C, Shi Z, et al. Caregiver burden and prevalence of depression, anxiety and sleep disturbances in Alzheimer's disease caregivers in China. *Journal of clinical nursing.* 2017;26(9-10):1291-1300.

9. Bargagli AM, Cascini S, Agabiti N, Kirchmayer U, Marino C, Davoli M. Determinants of antipsychotic drugs prescription among community-living older adults with dementia: A population-based study using health information systems in the Lazio Region, Italy. *Clinical interventions in aging.* 2019;14:2071-2083.

10. Reed C, Belger M, Scott Andrews J, et al. Factors associated with long-term impact on informal caregivers during Alzheimer's disease dementia progression: 36-month results from GERAS. *International psychogeriatrics.* 2019.

11. O'Shea E, Hopper L, Marques M, et al. A comparison of self and proxy quality of life ratings for people with dementia and their carers: a European prospective cohort study. *Aging & mental health.* 2020;24(1):162-170.

12. Trammell AR, McDaniel DJ, Obideen M, et al. Perceived Stress is Associated with Alzheimer's Disease Cerebrospinal Fluid Biomarkers in African Americans with Mild Cognitive Impairment. *Journal of Alzheimer's disease : JAD.* 2020;77(2):843-853.

13. Victor CR, Rippon I, Nelis SM, et al. Prevalence and determinants of loneliness in people living with dementia: Findings from the IDEAL programme. *International journal of geriatric psychiatry.* 2020;35(8):851-858.

**3) not community setting: 17**

1. Gambassi G, Lapane KL, Landi F, Sgadari A, Mor V, Bernabei R. Gender differences in the relation between comorbidity and mortality of patients with Alzheimer's disease. *Neurology.* 1999;53(3):508-516.

2. Barak Y, Aizenberg D. Suicide amongst Alzheimer's disease patients: a 10-year survey. *Dementia and geriatric cognitive disorders.* 2002;14(2):101-103.

3. Shah A, Ellanchenny N, Suh GH. A comparative study of behavioral and psychological signs and symptoms of dementia in patients with dementia referred to psychogeriatric services in Korea and the United Kingdom. *International psychogeriatrics.* 2004;16(2):219-236.

4. López-Pousa S, Vilalta-Franch J, Garre-Olmo J, Pons S, Cucurella MG. [Characterisation and prevalence of the psychological and behavioural symptoms in patients with dementia]. *Revista de neurologia.* 2007;45(11):683-688.

5. Nobili A, Pasina L, Trevisan S, et al. Use and misuse of antipsychotic drugs in patients with dementia in Alzheimer special care units. *International clinical psychopharmacology.* 2009;24(2):97-104.

6. Starkstein SE, Dragovic M, Jorge R, Brockman S, Robinson RG. Diagnostic criteria for depression in Alzheimer disease: a study of symptom patterns using latent class analysis. *The American journal of geriatric psychiatry : official journal of the American Association for Geriatric Psychiatry.* 2011;19(6):551-558.

7. Bergh S, Holmen J, Saltvedt I, Tambs K, Selbæk G. Dementia and neuropsychiatric symptoms in nursing-home patients in Nord-Trøndelag County. *Tidsskrift for den Norske laegeforening : tidsskrift for praktisk medicin, ny raekke.* 2012;132(17):1956-1959.

8. Selbæk G, Engedal K. Stability of the factor structure of the Neuropsychiatric Inventory in a 31-month follow-up study of a large sample of nursing-home patients with dementia. *International psychogeriatrics.* 2012;24(1):62-73.

9. Choi J, Myung W, Chung JW, et al. Association between functional impairment, depression, and extrapyramidal signs in neuroleptic-free patients with Alzheimer disease. *Journal of geriatric psychiatry and neurology.* 2013;26(3):144-150.

10. Selbaek G, Engedal K, Benth J, Bergh S. The course of neuropsychiatric symptoms in nursing-home patients with dementia over a 53-month follow-up period. *International psychogeriatrics.* 2014;26(1):81-91.

11. Turró-Garriga O, Conde-Sala JL, Reñé-Ramírez R, López-Pousa S, Gascón-Bayarri J, Garre-Olmo J. [Prevalence of anosognosia in Alzheimer's disease]. *Medicina clinica.* 2014;143(1):13-19.

12. Giebel CM, Sutcliffe C, Renom-Guiteras A, et al. Depressive symptomatology in severe dementia in a European sample: prevalence, associated factors and prescription rate of antidepressants. *International psychogeriatrics.* 2015;27(4):657-667.

13. van der Spek K, Gerritsen DL, Smalbrugge M, et al. Only 10% of the psychotropic drug use for neuropsychiatric symptoms in patients with dementia is fully appropriate. The PROPER I-study. *International psychogeriatrics.* 2016;28(10):1589-1595.

14. Hodges JR, Piguet O, Linszen MMJ, et al. Understanding hallucinations in probable Alzheimer's disease: Very low prevalence rates in a tertiary memory clinic. *Annals of neurology.* 2018;10:358-362.

15. Halpern R, Seare J, Tong J, Hartry A, Olaoye A, Aigbogun MS. Using electronic health records to estimate the prevalence of agitation in Alzheimer disease/dementia. *International journal of geriatric psychiatry.* 2019;34(3):420-431.

16. Koopmans R, Garcia-Segura ME, Fischer CE, Schweizer TA, Munoz DG. APOE ɛ4/ɛ4 Is Associated with Aberrant Motor Behavior Through Both Lewy Body and Cerebral Amyloid Angiopathy Pathology in High Alzheimer's Disease Pathological Load. *Aging & mental health.* 2019;72(4):1077-1087.

17. Nagahama Y, Fukui T, Akutagawa H, et al. Prevalence and Clinical Implications of the Mirror and TV Signs in Advanced Alzheimer's Disease and Dementia with Lewy Bodies. *Dementia and geriatric cognitive disorders extra.* 2020:56-62.

**4) unclear study setting: 39**

1. Rockwell E, Jackson E, Vilke G, Jeste DV. A Study of Delusions in a Large Cohort of Alzheimer's Disease Patients. *The American journal of geriatric psychiatry : official journal of the American Association for Geriatric Psychiatry.* 1994;2(2):157-164.

2. Chemerinski E, Petracca G, Manes F, Leiguarda R, Starkstein SE. Prevalence and correlates of anxiety in Alzheimer's disease. *Depression and anxiety.* 1998;7(4):166-170.

3. Weiner MF, Doody RS, Sairam R, Foster B, Liao TY. Prevalence and incidence of major depressive disorder in Alzheimer's disease: Findings from two databases. *Dementia and geriatric cognitive disorders.* 2002;13(1):8-12.

4. Lechowski L, Dieudonné B, Tortrat D, et al. Role of behavioural disturbance in the loss of autonomy for activities of daily living in Alzheimer patients. *International journal of geriatric psychiatry.* 2003;18(11):977-982.

5. Lopez OL, Becker JT, Sweet RA, et al. Psychiatric symptoms vary with the severity of dementia in probable Alzheimer's disease. *The Journal of neuropsychiatry and clinical neurosciences.* 2003;15(3):346-353.

6. Senanarong V, Cummings JL, Fairbanks L, et al. Agitation in Alzheimer's disease is a manifestation of frontal lobe dysfunction. *Dementia and geriatric cognitive disorders.* 2004;17(1-2):14-20.

7. Bacanu SA, Devlin B, Chowdari KV, DeKosky ST, Nimgaonkar VL, Sweet RA. Heritability of psychosis in Alzheimer disease. *The American journal of geriatric psychiatry : official journal of the American Association for Geriatric Psychiatry.* 2005;13(7):624-627.

8. Engelborghs S, Maertens K, Nagels G, et al. Neuropsychiatric symptoms of dementia: cross-sectional analysis from a prospective, longitudinal Belgian study. *International journal of geriatric psychiatry.* 2005;20(11):1028-1037.

9. Starkstein SE, Jorge R, Mizrahi R, Robinson RG. The construct of minor and major depression in Alzheimer's disease. *American Journal of Psychiatry.* 2005;162(11):2086-2093.

10. Starkstein SE, Jorge R, Mizrahi R, Robinson RG. A prospective longitudinal study of apathy in Alzheimer's disease. *Journal of neurology, neurosurgery, and psychiatry.* 2006;77(1):8-11.

11. Starkstein SE, Jorge R, Mizrahi R, Robinson RG. A diagnostic formulation for anosognosia in Alzheimer's disease. *Journal of neurology, neurosurgery, and psychiatry.* 2006;77(6):719-725.

12. Aarsland D, Brønnick K, Ehrt U, et al. Neuropsychiatric symptoms in patients with Parkinson's disease and dementia: frequency, profile and associated care giver stress. *Journal of neurology, neurosurgery, and psychiatry.* 2007;78(1):36-42.

13. Schulz R, McGinnis KA, Zhang S, et al. Dementia patient suffering and caregiver depression. *Alzheimer disease and associated disorders.* 2008;22(2):170-176.

14. Agüera-Ortiz L, Frank-García A, Gil P, Moreno A. Clinical progression of moderate-to-severe Alzheimer's disease and caregiver burden: a 12-month multicenter prospective observational study. *International psychogeriatrics.* 2010;22(8):1265-1279.

15. Castilla-Puentes RC, Habeych ME. Subtypes of depression among patients with Alzheimer's disease and other dementias. *Alzheimer's & dementia : the journal of the Alzheimer's Association.* 2010;6(1):63-69.

16. Vilalta-Franch J, López-Pousa S, Turon-Estrada A, et al. Syndromic association of behavioral and psychological symptoms of dementia in Alzheimer disease and patient classification. *The American journal of geriatric psychiatry : official journal of the American Association for Geriatric Psychiatry.* 2010;18(5):421-432.

17. Bliwise DL, Mercaldo ND, Avidan AY, Boeve BF, Greer SA, Kukull WA. Sleep disturbance in dementia with Lewy bodies and Alzheimer's disease: a multicenter analysis. *Dementia and geriatric cognitive disorders.* 2011;31(3):239-246.

18. Proitsi P, Hamilton G, Tsolaki M, et al. A Multiple Indicators Multiple Causes (MIMIC) model of Behavioural and Psychological Symptoms in Dementia (BPSD). *Neurobiology of aging.* 2011;32(3):434-442.

19. Benoit M, Berrut G, Doussaint J, et al. Apathy and depression in mild Alzheimer's disease: a cross-sectional study using diagnostic criteria. *Journal of Alzheimer's disease : JAD.* 2012;31(2):325-334.

20. Chiu PY, Steffens D, Chen PK, Hsu YC, Huang HT, Lai TJ. Depression in Taiwanese patients with Alzheimer's disease determined by the National Institutes of Mental Health Provisional Criteria. *International psychogeriatrics.* 2012;24(8):1299-1305.

21. Cohen CI, McKenzie SE, Rahmani M, Singh J, Prospere E. Historical changes in the severity of dementia and accompanying neuropsychiatric symptoms in persons presenting for evaluation in a multiracial urban dementia center. *Alzheimer disease and associated disorders.* 2012;26(4):352-357.

22. D'Onofrio G, Sancarlo D, Panza F, et al. Neuropsychiatric symptoms and functional status in Alzheimer's disease and vascular dementia patients. *Current Alzheimer research.* 2012;9(6):759-771.

23. Van der Mussele S, Bekelaar K, Le Bastard N, et al. Prevalence and associated behavioral symptoms of depression in mild cognitive impairment and dementia due to Alzheimer's disease. *International journal of geriatric psychiatry.* 2013;28(9):947-958.

24. Van der Mussele S, Le Bastard N, Vermeiren Y, et al. Behavioral symptoms in mild cognitive impairment as compared with Alzheimer's disease and healthy older adults. *International journal of geriatric psychiatry.* 2013;28(3):265-275.

25. Vilalta-Franch J, Calvó-Perxas L, Garre-Olmo J, Turró-Garriga O, López-Pousa S. Apathy syndrome in Alzheimer's disease epidemiology: prevalence, incidence, persistence, and risk and mortality factors. *Journal of Alzheimer's disease : JAD.* 2013;33(2):535-543.

26. Knapskog AB, Barca ML, Engedal K. Prevalence of depression among memory clinic patients as measured by the Cornell Scale of Depression in Dementia. *Aging & mental health.* 2014;18(5):579-587.

27. Van der Mussele S, Mariën P, Saerens J, et al. Behavioral syndromes in mild cognitive impairment and Alzheimer's disease. *Journal of Alzheimer's disease : JAD.* 2014;38(2):319-329.

28. Lou Q, Liu S, Huo YR, Liu M, Liu S, Ji Y. Comprehensive analysis of patient and caregiver predictors for caregiver burden, anxiety and depression in Alzheimer's disease. *Journal of clinical nursing.* 2015;24(17-18):2668-2678.

29. Van der Mussele S, Le Bastard N, Saerens J, et al. Agitation-associated behavioral symptoms in mild cognitive impairment and Alzheimer's dementia. *Aging & mental health.* 2015;19(3):247-257.

30. Van der Mussele S, Mariën P, Saerens J, et al. Psychosis associated behavioral and psychological signs and symptoms in mild cognitive impairment and Alzheimer's dementia. *Aging & mental health.* 2015;19(9):818-828.

31. Garre-Olmo J, Vilalta-Franch J, Calvó-Perxas L, Turró-Garriga O, Conde-Sala L, López-Pousa S. A path analysis of patient dependence and caregiver burden in Alzheimer's disease. *International psychogeriatrics.* 2016;28(7):1133-1141.

32. Thyrian JR, Eichler T, Reimann M, et al. Depressive symptoms and depression in people screened positive for dementia in primary care - results of the DelpHi-study. *International psychogeriatrics.* 2016;28(6):929-937.

33. Finger E, Zhang J, Dickerson B, Bureau Y, Masellis M. Disinhibition in Alzheimer's Disease is Associated with Reduced Right Frontal Pole Cortical Thickness. *Journal of Alzheimer's disease : JAD.* 2017;60(3):1161-1170.

34. Kabeshita Y, Adachi H, Matsushita M, et al. Sleep disturbances are key symptoms of very early stage Alzheimer disease with behavioral and psychological symptoms: a Japan multi-center cross-sectional study (J-BIRD). *International journal of geriatric psychiatry.* 2017;32(2):222-230.

35. Kumfor F, Landin-Romero R, Hazelton J, et al. Prevalence and neurobiological basis of delusions in dementia. *Journal of the neurological sciences.* 2017;381:675.

36. Salazar R, Dwivedi AK, Royall DR. Cross-Ethnic Differences in the Severity of Neuropsychiatric Symptoms in Persons With Mild Cognitive Impairment and Alzheimer's Disease. *The Journal of neuropsychiatry and clinical neurosciences.* 2017;29(1):13-21.

37. Sennik S, Schweizer TA, Fischer CE, Munoz DG. Risk Factors and Pathological Substrates Associated with Agitation/Aggression in Alzheimer's Disease: A Preliminary Study using NACC Data. *Journal of Alzheimer's disease : JAD.* 2017;55(4):1519-1528.

38. Chen P, Guarino PD, Dysken MW, et al. Neuropsychiatric Symptoms and Caregiver Burden in Individuals With Alzheimer's Disease: The TEAM-AD VA Cooperative Study. *Journal of geriatric psychiatry and neurology.* 2018;31(4):177-185.

39. Choi JW, Lee KS, Han E. Suicide risk within 1 year of dementia diagnosis in older adults: a nationwide retrospective cohort study. *Journal of psychiatry & neuroscience : JPN.* 2020;46(1):190219.

**5) Classification between participants in community, nursing home, and/or hospital is not clear: 14**

1. Newman SC. The prevalence of depression in Alzheimer's disease and vascular dementia in a population sample. *Journal of affective disorders.* 1999;52(1-3):169-176.

2. Craig D, Mirakhur A, Hart DJ, McIlroy SP, Passmore AP. A cross-sectional study of neuropsychiatric symptoms in 435 patients with Alzheimer's disease. *The American journal of geriatric psychiatry : official journal of the American Association for Geriatric Psychiatry.* 2005;13(6):460-468.

3. Hollingworth P, Hamshere ML, Moskvina V, et al. Four components describe behavioral symptoms in 1,120 individuals with late-onset Alzheimer's disease. *Journal of the American Geriatrics Society.* 2006;54(9):1348-1354.

4. Starkstein SE, Jorge R, Mizrahi R. The prevalence, clinical correlates and treatment of apathy in Alzheimer's disease. *European Journal of Psychiatry.* 2006;20(2):96-106.

5. Porta-Etessam J, Tobaruela-González JL, Rabes-Berendes C. Depression in patients with moderate Alzheimer disease: a prospective observational cohort study. *Alzheimer disease and associated disorders.* 2011;25(4):317-325.

6. Xing Y, Wei C, Chu C, et al. Stage-specific gender differences in cognitive and neuropsychiatric manifestations of vascular dementia. *American journal of Alzheimer's disease and other dementias.* 2012;27(6):433-438.

7. Truzzi A, Ulstein I, Valente L, et al. Patterns of neuropsychiatric sub-syndromes in Brazilian and Norwegian patients with dementia. *International psychogeriatrics.* 2013;25(2):228-235.

8. Steinberg M, Hess K, Corcoran C, et al. Vascular risk factors and neuropsychiatric symptoms in Alzheimer's disease: the Cache County Study. *International journal of geriatric psychiatry.* 2014;29(2):153-159.

9. Snowden MB, Atkins DC, Steinman LE, et al. Longitudinal Association of Dementia and Depression. *The American journal of geriatric psychiatry : official journal of the American Association for Geriatric Psychiatry.* 2015;23(9):897-905.

10. Liao W, Hamel RE, Olde Rikkert MG, et al. A profile of The Clinical Course of Cognition and Comorbidity in Mild Cognitive Impairment and Dementia Study (The 4C study): two complementary longitudinal, clinical cohorts in the Netherlands. *BMC neurology.* 2016;16(1):242.

11. Maust DT, Langa KM, Blow FC, Kales HC. Psychotropic use and associated neuropsychiatric symptoms among patients with dementia in the USA. *International journal of geriatric psychiatry.* 2017;32(2):164-174.

12. FitzGerald JM, Perera G, Chang-Tave A, et al. The Incidence of Recorded Delirium Episodes Before and After Dementia Diagnosis: Differences Between Dementia With Lewy Bodies and Alzheimer's Disease. *Journal of the American Medical Directors Association.* 2019;20(5):604-609.

13. Manso-Calderón R. The impact of vascular burden on behavioural and psychological symptoms in older adults with dementia: the BEVASDE study. *JIMD reports.* 2020;41(1):165-174.

14. Parrotta I, De Mauleon A, Abdeljalil AB, et al. Depression in People With Dementia and Caregiver Outcomes: Results From the European Right Time Place Care Study. *Journal of the American Medical Directors Association.* 2020;21(6):872-878.e871.

**6) no details (conference abstract): 46**

1. Chung CS, Lee J, Shin W, et al. Characteristics of delusions in Alzheimer's dementia patients. *Alzheimer's and Dementia.* 2009;5(4):191.

2. Rajender G, Sengupta S, Bala K, Kanwal K. Study of BPSD in Vascular dementia and Alzheimer's disease. *Indian journal of psychiatry.* 2009;51(6):S139-S140.

3. Ribeiz SRI, Bassitt DP, Litvoc J, Bottino CMC. Psychotic symptoms in the elderly: Prevalence in Alzheimer's disease and nondemented individuals from a community-based sample in Brazil. *Alzheimer's and Dementia.* 2009;5(4):296.

4. Bozoglu E, Naharci I, Doruk H, Isik AT. Evaluation of prevalence and treatment of neuropsychiatric symptoms in elderly patients with dementia: 9 year-follow-up. *Alzheimer's and Dementia.* 2011;7(4):S239.

5. Lacey L, Niecko T, Leibman C, Liu E. Relationship between patient dependence on others and clinical measures of cognition, function and behavior in Alzheimer's disease (AD): Results from a longitudinal study. *Alzheimer's and Dementia.* 2011;7(4):S644.

6. Vogel A, Bhattacharya S, Waldorff F, Waldemar G. Proxy-rated quality of life in alzheimer's disease: A 3-year longitudinal study. *Alzheimer's and Dementia.* 2011;7(4):S641.

7. Donovan N, Wadsworth L, Lorius N, et al. Regional cortical thinning predicts worsening apathyand hallucinations in mild cognitive impairment and mild Alzheimer's disease. *Alzheimer's and Dementia.* 2012;8(4):P26.

8. Koller EA, Adler GS. Exploration of the interaction between diabetes, cardiovascular disease, depression, and dementia in the community-dwelling medicare beneficiary population. *Endocrine Reviews.* 2012;33(3).

9. Sadak T, Katon J, Borson S. Multimorbidity in Alzheimer's disease and related dementias. *Alzheimer's and Dementia.* 2012;8(4):P490.

10. Sadak T, Kukull W, Borson S, Logsdon R, Cochrane B, Herting J. Multidimensional complexity in the dementias: Diagnosis, stage, comorbidity, and neuropsychiatric symptoms. *Neurology.* 2012;78(1).

11. Van Der Linde RM, Stephan BCM, Savva GM, Dening T, Brayne C. Longitudinal course of behavioural and psychological symptoms of dementia. *American journal of epidemiology.* 2012;175:S1.

12. Gon¸alves-Pereira M, Cardoso AM, Verdelho A, et al. The 10/66-dementia research group prevalence study in Portugal: Preliminary results (2012). *Psychotherapy and Psychosomatics.* 2013;82:37.

13. Kang HS, Noh YJ, Shim SH, Back JS, Kim DK. Factors associated with caregiver burden in patients with Alzheimer's disease. *International psychogeriatrics.* 2013;25:S92.

14. Yoon H, Choi H. Association between insight and neuropsychiatric symptoms in Alzheimer's dementia: A CREDOS (Clinical Research Center for Dementia of South Korea) Study. *International psychogeriatrics.* 2013;25:S144.

15. Arai H, Kobayashi H, Taguchi M, Yamauchi K, Nakamura Y. A prospective cohort study on mortality risk associated with atypical antipsychotics in patients with Alzheimer's disease: Japan consortium for antipsychotics treatment in Alzheimer's disease (J-Catia). *Alzheimer's and Dementia.* 2014;10:P530.

16. Cho ST, Jung HB, Lee YG, Kim KK, Na HR. Correlation between overactive bladder symptom score (OABSS) and neuropsychological parameters in Alzheimer's disease patients with lower urinary tract symptom. *Journal of Urology.* 2014;191(4):e944.

17. Ramdane S. The profile of neuropsychiatric symptoms across dementia stages in Alzheimer's disease: An observational study. *Alzheimer's and Dementia.* 2014;10:P524-P525.

18. Stefanova E, Stojmenovic GM, Stojkovic T, et al. Distinct neuropsychological and behavioural profiles in early-onset Alzheimer's disease: Report from belgrade memory clinic. *Alzheimer's and Dementia.* 2014;10:P179.

19. Francisco EPE, Tay RY, Hum AYM, et al. Symptom burden and quality of life of advanced dementia patients in a home care setting-a cross-sectional study. *Annals of the Academy of Medicine Singapore.* 2015;44(10):S140.

20. Mayer S, Granvik E, Minthon L, Nägga K. Prevalence, course and treatment of neuropsychiatric symptoms in cases with severe dementia-a 4-year update of the swedish BPSD-registry. *Neurodegenerative Diseases.* 2015;15:1904.

21. Thyrian JR, Eichler T, Teipel SJ, Hoffmann W. Depressive symptoms and depression in people screened positive for dementia. *Alzheimer's and Dementia.* 2015;11(7):P829.

22. Winblad B, Aarsland D, Kramberger MG, Abdelnour C, Olmo JG, Lemstra E. Visual hallucinations and rate of cognitive decline in DLB. A longitudinal multicenter cohort study (E-DLB). *American Journal of Neurodegenerative Diseases.* 2015;4:63.

23. Castro FA, Melgarejo JD, Lee JH, Maestre GE. Neuropsychiatric symptoms and their relationship with progression to severe dementia and death: Findings of the maracaibo aging study (MAS). *Alzheimer's and Dementia.* 2016;12(7):P495-P496.

24. Guartazaca Gerrero EP, Llibre Guerra JJ, Llibre JC, Hernández MAG. Behavioral and psychological symptoms in elderly patients with dementia: A 5-year cohort follow up. *Alzheimer's and Dementia.* 2016;12(7):P487.

25. Kwon JC, Kim B, Lee K, Choi NC, Jung Y. The comparisons of etiologies, clinical features focusing on behavior problem and caregiver burdens between early onset dementia (EOD) and late onset dementia (LOD). *Alzheimer's and Dementia.* 2016;12(7):P587.

26. Llibre-Guerra JJ, Llibre-Rodriguez JJ, Guerra-Hernández MA, et al. Behavioral and psychological symptoms in elderly patients with dementia A 5 years cohort follow up. *International Journal of Psychophysiology.* 2016;108:110.

27. Maxwell CJ, Mondor L, Hogan DB, et al. The relative impact of multimorbidity on health-related quality of life: A comparison of persons with and without dementia. *Alzheimer's and Dementia.* 2016;12(7):P1013.

28. Multani N, Rudzicz F, Tartaglia MC. Comparing neuropsychiatric and language features in early-onset and late-onset Alzheimer's disease. *Alzheimer's and Dementia.* 2016;12(7):P490.

29. Tay RY, Erwin PF, Mervyn YHK, et al. Symptom prevalence and quality of life of advanced dementia patients in a homecare setting: A prospective cross-sectional study. *Palliative medicine.* 2016;30(6):NP223-NP224.

30. Aigbogun MS, Stellhorn RA, Hartry A, Hammer-Helmich L, Baker R, Fillit HM. Prevalence of behavioral disturbances and associated burden in patients with dementia in us claims data: Is there under-reporting? *Value in Health.* 2017;20(5):A188.

31. Creese B, Ballard C, Aarsland D, Bergh S, Rongve A, Selbaek G. An evaluation of predictors and cognitive decline associated with persistent and transient psychotic symptoms in Alzheimer's disease. *Alzheimer's and Dementia.* 2017;13(7):P367.

32. Ehrenberg AJ, Suemoto CK, Petersen C, et al. The contribution of hyperphosphorylated-tau pathology to neuropsychiatric symptoms in Alzheimer's disease. *Alzheimer's and Dementia.* 2017;13(7):P1142-P1144.

33. Johar I, Aarsland D, Ballard C, et al. Prevalence and co-morbidity of apathy and depression in Alzheimer's disease. *Alzheimer's and Dementia.* 2017;13(7):P371-P372.

34. Kwon JC, Lee K. Comparisons of etiologies, clinical features focusing on behavior problems and caregiver burdens between early-onset dementia (EOD) and late-onset dementia (LOD). *Alzheimer's and Dementia.* 2017;13(7):P829.

35. Ortiz LA, Castillo-García I. Pathways of neuropsychiatric symptoms until death in moderate to severe dementia. *Alzheimer's and Dementia.* 2017;13(7):P716.

36. Salgado AR, Llibre J. Behavioral and psychological symptoms in elderly patients with dementia: A 5 years cohort follow up. *Journal of Neuropsychiatry and Clinical Neurosciences.* 2017;29(3):e23.

37. Siafarikas N, Fladby T, Selbaek G, Saltyte Benth J, Auning E, Aarsland D. Neuropsychiatric symptoms in mild cognitive impairment and different stages of Alzheimer's dementia. *Neurodegenerative Diseases.* 2017;17:1778-1780.

38. Atayde AL, Fischer CE, Schweizer TA, Munoz DG. ASSOCIATION OF DECLINING COGNITIVE STATUS AND NIGHTTIME BEHAVIOUR PREVALENCE ALONG THE SPECTRUM OF ALZHEIMER'S DISEASE SEVERITY. *Alzheimer's and Dementia.* 2018;14(7):P407.

39. Black CM, Lipton RB, Thiel E, Brouillette M, Khandker RK. PREVALENCE OF DIAGNOSED SYMPTOMS PRECEDING ALZHEIMER'S DISEASE DIAGNOSIS AMONG TREATED AND UNTREATED ALZHEIMER'S DISEASE PATIENTS WITH COMMERCIAL AND MEDICARE SUPPLEMENTAL INSURANCE. *Alzheimer's and Dementia.* 2018;14(7):P412.

40. Mirza SS, Sapkota S, Ramirez J, et al. NEUROPSYCHIATRIC SYMPTOMS PREDICT DAILY LIFE FUNCTIONALITY IN DEMENTIA PATIENTS: RESULTS FROM THE SUNNYBROOK DEMENTIA STUDY. *Alzheimer's and Dementia.* 2018;14(7):P1558.

41. Nunez NA, Yin S, McCarthy J, Ducharme S. A COMPARATIVE STUDY EXAMINING PREVALENCE OF LIFETIME PSYCHIATRIC DISORDERS AS A RISK FACTOR FOR BEHAVIORAL VARIANT FRONTOTEMPORAL DEMENTIA AND ALZHEIMER'S DISEASE. *Alzheimer's and Dementia.* 2018;14(7):P657.

42. Aigbogun MS, Jones E, Husbands J, et al. BEHAVIORAL SYMPTOMS AND DISTURBANCES IN DEMENTIA: A HIGHLY PREVALENT CONDITION. *Alzheimer's and Dementia.* 2019;15(7):P356.

43. Cuccaro ML, Celis K, Adams LD, et al. NEUROPSYCHIATRIC FEATURES IN AFRICAN AMERICAN (AA) AND CAUCASIAN (CA) INDIVIDUALS WITH ALZHEIMER DISEASE (AD). *Alzheimer's and Dementia.* 2019;15(7):P1056.

44. Da Silva MV, Creese B, Johar I, et al. THE COURSE OF APATHY IN PEOPLE WITH DEMENTIA. *Alzheimer's and Dementia.* 2019;15(7):P570.

45. Mirzaei F, Huang E, Wang N, Li L, Krzywy H, Ratti E. INSIGHT INTO THE FRONTOTEMPORAL DEMENTIA (FTD) PATIENTS’ JOURNEY THROUGH THE U.S. HEALTHCARE SYSTEM: AN ELECTRONIC HEALTH RECORD ANALYSIS. *Alzheimer's and Dementia.* 2019;15(7):P479.

46. Sibley A, Shrestha S, Lipovac-Dew M, Kunik M. ADDRESSING DEPRESSION WITH AND WITHOUT COEXISTING ANXIETY IN COMMUNITY-DWELLING PERSONS WITH DEMENTIA. *American Journal of Geriatric Psychiatry.* 2020;28(4):S84-S85.

**7) dementia patients less than 300: 33**

1. Burke WJ, Rubin EH, Morris JC, Berg L. Symptoms of "depression" in dementia of the Alzheimer type. *Alzheimer disease and associated disorders.* 1988;2(4):356-362.

2. Rubin EH, Drevets WC, Burke WJ. The nature of psychotic symptoms in senile dementia of the Alzheimer type. *Journal of geriatric psychiatry and neurology.* 1988;1(1):16-20.

3. Cooper JK, Mungas D, Verma M, Weiler PG. Psychotic symptoms in Alzheimer's disease. *International journal of geriatric psychiatry.* 1991;6(10):721-726.

4. Skoog I. The prevalence of psychotic, depressive and anxiety syndromes in demented and non-demented 85-year-olds. *International journal of geriatric psychiatry.* 1993;8(3):247-253.

5. Allen H, Jolley D, Comish J, Burns A. Depression in dementia: a study of mood in a community sample and referrals to a community service. *International journal of geriatric psychiatry.* 1997;12(5):513-518.

6. Ballard CG, O'Brien JT, Coope B, Wilcock G. Psychotic symptoms in dementia and the rate of cognitive decline. *Journal of the American Geriatrics Society.* 1997;45(8):1031-1032.

7. Aevarsson O, Svanborg A, Skoog I. Seven-year survival rate after age 85 years: relation to Alzheimer disease and vascular dementia. *Archives of neurology.* 1998;55(9):1226-1232.

8. Ballard C, McKeith IG. Psychiatric features in diffuse Lewy body disease. *Neurology.* 1998;50(2):573.

9. Aarsland D, Cummings JL, Larsen JP. Neuropsychiatric differences between Parkinson's disease with dementia and Alzheimer's disease. *International journal of geriatric psychiatry.* 2001;16(2):184-191.

10. Chow TW, Liu CK, Fuh JL, et al. Neuropsychiatric symptoms of Alzheimer's disease differ in Chinese and American patients. *International journal of geriatric psychiatry.* 2002;17(1):22-28.

11. Hart DJ, Craig D, Compton SA, et al. A retrospective study of the behavioural and psychological symptoms of mid and late phase Alzheimer's disease. *International journal of geriatric psychiatry.* 2003;18(11):1037-1042.

12. Kao AW, Racine CA, Quitania LC, Kramer JH, Christine CW, Miller BL. Cognitive and neuropsychiatric profile of the synucleinopathies: Parkinson disease, dementia with Lewy bodies, and multiple system atrophy. *Alzheimer disease and associated disorders.* 2009;23(4):365-370.

13. Savva GM, Zaccai J, Matthews FE, Davidson JE, McKeith I, Brayne C. Prevalence, correlates and course of behavioural and psychological symptoms of dementia in the population. *The British journal of psychiatry : the journal of mental science.* 2009;194(3):212-219.

14. Shaji KS, George RK, Prince MJ, Jacob KS. Behavioral symptoms and caregiver burden in dementia. *Indian journal of psychiatry.* 2009;51(1):45-49.

15. Meagher DJ, Leonard M, Donnelly S, Conroy M, Saunders J, Trzepacz PT. A comparison of neuropsychiatric and cognitive profiles in delirium, dementia, comorbid delirium-dementia and cognitively intact controls. *Journal of neurology, neurosurgery, and psychiatry.* 2010;81(8):876-881.

16. Koppel J, Sunday S, Buthorn J, Goldberg T, Davies P, Greenwald B. Elevated CSF Tau is associated with psychosis in Alzheimer's disease. *The American journal of psychiatry.* 2013;170(10):1212-1213.

17. Trzepacz PT, Yu P, Bhamidipati PK, et al. Frontolimbic atrophy is associated with agitation and aggression in mild cognitive impairment and Alzheimer's disease. *Alzheimer's & dementia : the journal of the Alzheimer's Association.* 2013;9(5 Suppl):S95-S104.e101.

18. Donovan NJ, Wadsworth LP, Lorius N, et al. Regional cortical thinning predicts worsening apathy and hallucinations across the Alzheimer disease spectrum. *The American journal of geriatric psychiatry : official journal of the American Association for Geriatric Psychiatry.* 2014;22(11):1168-1179.

19. Fonseca L, Machado Á. Suicidal behaviour in frontotemporal dementia patients--a retrospective study. *International journal of geriatric psychiatry.* 2014;29(2):217-218.

20. Bertoux M, de Souza LC, Sarazin M, Funkiewiez A, Dubois B, Hornberger M. How Preserved is Emotion Recognition in Alzheimer Disease Compared With Behavioral Variant Frontotemporal Dementia? *Alzheimer disease and associated disorders.* 2015;29(2):154-157.

21. Enache D, Cavallin L, Lindberg O, et al. Medial temporal lobe atrophy and depressive symptoms in elderly patients with and without Alzheimer disease. *Journal of geriatric psychiatry and neurology.* 2015;28(1):40-48.

22. Gatchel JR, Donovan NJ, Locascio JJ, et al. Regional 18F-Fluorodeoxyglucose Hypometabolism is Associated with Higher Apathy Scores Over Time in Early Alzheimer Disease. *Alzheimer's research & therapy.* 2017;25(7):683-693.

23. Kim WH, Kim JH, Kim BS, et al. The role of depression in the insomnia of people with subjective memory impairment, mild cognitive impairment, and dementia in a community sample of elderly individuals in South Korea. *International psychogeriatrics.* 2017;29(4):653-661.

24. Kunschmann R, Busse S, Frodl T, Busse M. Psychotic Symptoms Associated with Poor Renal Function in Mild Cognitive Impairment and Dementias. *Journal of Alzheimer's disease : JAD.* 2017;58(1):243-252.

25. Maust DT, Kales HC, McCammon RJ, Blow FC, Leggett A, Langa KM. Distress Associated with Dementia-Related Psychosis and Agitation in Relation to Healthcare Utilization and Costs. *The American journal of geriatric psychiatry : official journal of the American Association for Geriatric Psychiatry.* 2017;25(10):1074-1082.

26. Eichler T, Thyrian JR, Hertel J, et al. Patient Variables Associated with the Assignment of a Formal Dementia Diagnosis to Positively Screened Primary Care Patients. *Current Alzheimer research.* 2018;15(1):44-50.

27. Kontogianni E, Skaltsounaki E, Politis A, et al. Sex Differences in the Neuropsychiatric Symptoms of Patients With Alzheimer's Disease. *Current gerontology and geriatrics research.* 2018;33(7):450-457.

28. D'Antonio F, Di Vita A, Zazzaro G, et al. Psychosis of Alzheimer's disease: Neuropsychological and neuroimaging longitudinal study. *International journal of geriatric psychiatry.* 2019;34(11):1689-1697.

29. Engedal K, Haugen PK, Dourado MCN, et al. Factors associated with sleep in family caregivers of individuals with dementia. *International journal of qualitative studies on health and well-being.* 2019;55(1):95-102.

30. Lim Chiong CJ, Miller AB, Lim Chiong CJ, Chiong J. PREVALENCE OF PSYCHOTROPIC MEDICATION USE FOR NEUROPSYCHIATRIC SYMPTOMS IN COMMUNITY DWELLING HOME BOUND DEMENTIA PATIENTS. *Alzheimer's and Dementia.* 2019;15(7):P460-P461.

31. Rozum WJ, Cooley B, Vernon E, Matyi J, Tschanz JT. Neuropsychiatric symptoms in severe dementia: Associations with specific cognitive domains the Cache County Dementia Progression Study. 2019;34(7):1087-1094.

32. Scaricamazza E, Colonna I, Sancesario GM, et al. Neuropsychiatric symptoms differently affect mild cognitive impairment and Alzheimer's disease patients: a retrospective observational study. 2019;40(7):1377-1382.

33. Stoner CR. The psychometric properties of the control, autonomy, self-realisation and pleasure scale (CASP-19) for older adults with dementia. *Aging & mental health.* 2019;23(5):643-649.

**8) present only symptom score or ratio values for BPSD: 79**

Note. This is when the average severity of BPSD symptoms in participants is presented only, not the prevalence, or the percentage of participants with BPSD, but the raw data cannot be clearly estimated due to ambiguous reports.

1. Cohen D, Eisdorfer C, Gorelick P, et al. Psychopathology associated with Alzheimer's disease and related disorders. *Journal of gerontology.* 1993;48(6):M255-260.

2. Meshefedjian G, McCusker J, Bellavance F, Baumgarten M. Factors associated with symptoms of depression among informal caregivers of demented elders in the community. *The Gerontologist.* 1998;38(2):247-253.

3. Wilson RS, Gilley DW, Bennett DA, Beckett LA, Evans DA. Hallucinations, delusions, and cognitive decline in Alzheimer's disease. *Journal of neurology, neurosurgery, and psychiatry.* 2000;69(2):172-177.

4. Whyte EM, Mulsant BH, Butters MA, et al. Cognitive and behavioral correlates of low vitamin B12 levels in elderly patients with progressive dementia. *The American journal of geriatric psychiatry : official journal of the American Association for Geriatric Psychiatry.* 2002;10(3):321-327.

5. Chan DC, Kasper JD, Black BS, Rabins PV. Prevalence and correlates of behavioral and psychiatric symptoms in community-dwelling elders with dementia or mild cognitive impairment: the Memory and Medical Care Study. *International journal of geriatric psychiatry.* 2003;18(2):174-182.

6. Covinsky KE, Newcomer R, Fox P, et al. Patient and caregiver characteristics associated with depression in caregivers of patients with dementia. *Journal of general internal medicine.* 2003;18(12):1006-1014.

7. Bassiony MM, Rosenblatt A, Baker A, et al. Falls and age in patients with Alzheimer's disease. *The Journal of nervous and mental disease.* 2004;192(8):570-572.

8. Ferri CP, Ames D, Prince M. Behavioral and psychological symptoms of dementia in developing countries. *International psychogeriatrics.* 2004;16(4):441-459.

9. López-Pousa S, Garre-Olmo J, Turon-Estrada A, et al. [Cost relation between severity of Alzheimer's disease and cognitive and functional impairment]. *Medicina clinica.* 2004;122(20):767-772.

10. Sambrook R, Herrmann N, Hébert R, et al. Canadian Outcomes Study in Dementia: study methods and patient characteristics. *Canadian journal of psychiatry Revue canadienne de psychiatrie.* 2004;49(7):417-427.

11. Sink KM, Covinsky KE, Newcomer R, Yaffe K. Ethnic differences in the prevalence and pattern of dementia-related behaviors. *Journal of the American Geriatrics Society.* 2004;52(8):1277-1283.

12. Roberson ED, Hesse JH, Rose KD, et al. Frontotemporal dementia progresses to death faster than Alzheimer disease. *Neurology.* 2005;65(5):719-725.

13. Herrmann N, Lanctôt KL, Sambrook R, et al. The contribution of neuropsychiatric symptoms to the cost of dementia care. *International journal of geriatric psychiatry.* 2006;21(10):972-976.

14. Ortiz F, Fitten LJ, Cummings JL, Hwang S, Fonseca M. Neuropsychiatric and behavioral symptoms in a community sample of Hispanics with Alzheimer's disease. *American journal of Alzheimer's disease and other dementias.* 2006;21(4):263-273.

15. Sink KM, Covinsky KE, Barnes DE, Newcomer RJ, Yaffe K. Caregiver characteristics are associated with neuropsychiatric symptoms of dementia. *Journal of the American Geriatrics Society.* 2006;54(5):796-803.

16. Vilalta-Franch J, Garre-Olmo J, López-Pousa S, et al. Comparison of different clinical diagnostic criteria for depression in Alzheimer disease. *The American journal of geriatric psychiatry : official journal of the American Association for Geriatric Psychiatry.* 2006;14(7):589-597.

17. Wilson RS, Tang Y, Aggarwal NT, et al. Hallucinations, cognitive decline, and death in Alzheimer's disease. *Neuroepidemiology.* 2006;26(2):68-75.

18. Zhu CW, Scarmeas N, Torgan R, et al. Longitudinal study of effects of patient characteristics on direct costs in Alzheimer disease. *Neurology.* 2006;67(6):998-1005.

19. Soto ME, Andrieu S, Cantet C, et al. Predictive value of rapid decline in mini mental state examination in clinical practice for prognosis in Alzheimer's disease. *Dementia and geriatric cognitive disorders.* 2008;26(2):109-116.

20. Fernández M, Gobartt AL, Balañá M. Behavioural symptoms in patients with Alzheimer's disease and their association with cognitive impairment. *BMC neurology.* 2010;10:87.

21. Gustavsson A, Jonsson L, Rapp T, et al. Differences in resource use and costs of dementia care between European countries: baseline data from the ICTUS study. *The journal of nutrition, health & aging.* 2010;14(8):648-654.

22. Riedel O, Heuser I, Klotsche J, Dodel R, Wittchen HU. Occurrence risk and structure of depression in Parkinson disease with and without dementia: results from the GEPAD Study. *Journal of geriatric psychiatry and neurology.* 2010;23(1):27-34.

23. Riedel O, Klotsche J, Spottke A, et al. Frequency of dementia, depression, and other neuropsychiatric symptoms in 1,449 outpatients with Parkinson's disease. *Journal of neurology.* 2010;257(7):1073-1082.

24. Staekenborg SS, Su T, van Straaten EC, et al. Behavioural and psychological symptoms in vascular dementia; differences between small- and large-vessel disease. *Journal of neurology, neurosurgery, and psychiatry.* 2010;81(5):547-551.

25. Bergvall N, Brinck P, Eek D, et al. Relative importance of patient disease indicators on informal care and caregiver burden in Alzheimer's disease. *International psychogeriatrics.* 2011;23(1):73-85.

26. Knopman DS, Weintraub S, Pankratz VS. Language and behavior domains enhance the value of the clinical dementia rating scale. *Alzheimer's & dementia : the journal of the Alzheimer's Association.* 2011;7(3):293-299.

27. Livney MG, Clark CM, Karlawish JH, et al. Ethnoracial differences in the clinical characteristics of Alzheimer's disease at initial presentation at an urban Alzheimer's disease center. *The American journal of geriatric psychiatry : official journal of the American Association for Geriatric Psychiatry.* 2011;19(5):430-439.

28. Rhee Y, Csernansky JG, Emanuel LL, Chang CG, Shega JW. Psychotropic medication burden and factors associated with antipsychotic use: An analysis of a population-based sample of community-dwelling older persons with dementia. *Journal of the American Geriatrics Society.* 2011;59(11):2100-2107.

29. Tschanz JT, Corcoran CD, Schwartz S, et al. Progression of cognitive, functional, and neuropsychiatric symptom domains in a population cohort with Alzheimer dementia: the Cache County Dementia Progression study. *The American journal of geriatric psychiatry : official journal of the American Association for Geriatric Psychiatry.* 2011;19(6):532-542.

30. Calvó-Perxas L, de Eugenio RM, Marquez-Daniel F, et al. Profile and variables related to antipsychotic consumption according to dementia subtypes. *International psychogeriatrics.* 2012;24(6):940-947.

31. Sourdet S, van Kan GA, Soto ME, et al. Prognosis of an abnormal one-leg balance in community-dwelling patients with Alzheimer's disease: a 2-year prospective study in 686 patients of the REAL.FR study. *Journal of the American Medical Directors Association.* 2012;13(4):407.e401-406.

32. Beaudreau SA, Kaci Fairchild J, Spira AP, Lazzeroni LC, O'Hara R. Neuropsychiatric symptoms, apolipoprotein E gene, and risk of progression to cognitive impairment, no dementia and dementia: the Aging, Demographics, and Memory Study (ADAMS). *International journal of geriatric psychiatry.* 2013;28(7):672-680.

33. Stella F, Forlenza OV, Laks J, et al. The Brazilian version of the Neuropsychiatric Inventory-Clinician rating scale (NPI-C): reliability and validity in dementia. *International psychogeriatrics.* 2013;25(9):1503-1511.

34. Trzepacz PT, Saykin A, Yu P, et al. Subscale validation of the neuropsychiatric inventory questionnaire: comparison of Alzheimer's disease neuroimaging initiative and national Alzheimer's coordinating center cohorts. *The American journal of geriatric psychiatry : official journal of the American Association for Geriatric Psychiatry.* 2013;21(7):607-622.

35. Vilalta-Franch J, López-Pousa S, Calvó-Perxas L, Garre-Olmo J. Psychosis of Alzheimer disease: prevalence, incidence, persistence, risk factors, and mortality. *The American journal of geriatric psychiatry : official journal of the American Association for Geriatric Psychiatry.* 2013;21(11):1135-1143.

36. Zahodne LB, Devanand DP, Stern Y. Coupled cognitive and functional change in Alzheimer's disease and the influence of depressive symptoms. *Journal of Alzheimer's disease : JAD.* 2013;34(4):851-860.

37. Haro JM, Kahle-Wrobleski K, Bruno G, et al. Analysis of burden in caregivers of people with Alzheimer's disease using self-report and supervision hours. *The journal of nutrition, health & aging.* 2014;18(7):677-684.

38. Kamiya M, Sakurai T, Ogama N, Maki Y, Toba K. Factors associated with increased caregivers' burden in several cognitive stages of Alzheimer's disease. *Geriatrics & gerontology international.* 2014;14 Suppl 2:45-55.

39. Kang HS, Myung W, Na DL, et al. Factors associated with caregiver burden in patients with Alzheimer's disease. *Psychiatry investigation.* 2014;11(2):152-159.

40. Vu M, Hogan DB, Patten SB, et al. A comprehensive profile of the sociodemographic, psychosocial and health characteristics of Ontario home care clients with dementia. *Chronic diseases and injuries in Canada.* 2014;34(2-3):132-144.

41. Barca ML, Engedal K, Selbaek G, et al. Confirmatory factor analysis of the Cornell scale for depression in dementia among patient with dementia of various degrees. *Journal of affective disorders.* 2015;188:173-178.

42. Borges G, Acosta I, Sosa AL. Suicide ideation, dementia and mental disorders among a community sample of older people in Mexico. *International journal of geriatric psychiatry.* 2015;30(3):247-255.

43. Brodaty H, Connors MH, Xu J, Woodward M, Ames D. The course of neuropsychiatric symptoms in dementia: a 3-year longitudinal study. *Journal of the American Medical Directors Association.* 2015;16(5):380-387.

44. Chung EJ, Babulal GM, Monsell SE, Cairns NJ, Roe CM, Morris JC. Clinical Features of Alzheimer Disease With and Without Lewy Bodies. *JAMA neurology.* 2015;72(7):789-796.

45. Kahle-Wrobleski K, Andrews JS, Belger M, et al. Clinical and Economic Characteristics of Milestones along the Continuum of Alzheimer's Disease: Transforming Functional Scores into Levels of Dependence. *The journal of prevention of Alzheimer's disease.* 2015;2(2):115-120.

46. Moretti R, Cavressi M, Tomietto P. Gait and apathy as relevant symptoms of subcortical vascular dementia. *American journal of Alzheimer's disease and other dementias.* 2015;30(4):390-399.

47. Orgeta V, Orrell M, Edwards RT, Hounsome B, Woods B. Self- and Carer-Rated Pain in People With Dementia: Influences of Pain in Carers. *Journal of pain and symptom management.* 2015;49(6):1042-1049.

48. Orgeta V, Orrell M, Hounsome B, Woods B. Self and carer perspectives of quality of life in dementia using the QoL-AD. *International journal of geriatric psychiatry.* 2015;30(1):97-104.

49. Park HK, Choi SH, Park SA, et al. Cognitive profiles and neuropsychiatric symptoms in Korean early-onset Alzheimer's disease patients: a CREDOS study. *Journal of Alzheimer's disease : JAD.* 2015;44(2):661-673.

50. Peters ME, Schwartz S, Han D, et al. Neuropsychiatric symptoms as predictors of progression to severe Alzheimer's dementia and death: the Cache County Dementia Progression Study. *The American journal of psychiatry.* 2015;172(5):460-465.

51. Zahodne LB, Ornstein K, Cosentino S, Devanand DP, Stern Y. Longitudinal relationships between Alzheimer disease progression and psychosis, depressed mood, and agitation/aggression. *The American journal of geriatric psychiatry : official journal of the American Association for Geriatric Psychiatry.* 2015;23(2):130-140.

52. Chiu PY, Tsai CT, Chen PK, Chen WJ, Lai TJ. Neuropsychiatric Symptoms in Parkinson's Disease Dementia Are More Similar to Alzheimer's Disease than Dementia with Lewy Bodies: A Case-Control Study. *PloS one.* 2016;11(4):e0153989.

53. Farré M, Haro JM, Kostov B, et al. Direct and indirect costs and resource use in dementia care: A cross-sectional study in patients living at home. *International journal of nursing studies.* 2016;55:39-49.

54. Giebel C, Sutcliffe C, Verbeek H, et al. Depressive symptomatology and associated factors in dementia in Europe: home care versus long-term care. *International psychogeriatrics.* 2016;28(4):621-630.

55. Godefroy O, Bakchine S, Verny M, Delabrousse-Mayoux JP, Roussel M. Characteristics of Alzheimer's Disease Patients with Severe Executive Disorders. *Journal of Alzheimer's Disease.* 2016;51(3):815-825.

56. Krolak-Salmon P, Roubaud C, Finne-Soveri H, et al. Evaluation of a mobile team dedicated to behavioural disorders as recommended by the Alzheimer Cooperative Valuation in Europe joint action: observational cohort study. *European journal of neurology.* 2016;23(5):979-988.

57. Ranasinghe KG, Rankin KP, Lobach IV, et al. Cognition and neuropsychiatry in behavioral variant frontotemporal dementia by disease stage. *Neurology.* 2016;86(7):600-610.

58. Tokuchi R, Hishikawa N, Sato K, et al. Differences between the behavioral and psychological symptoms of Alzheimer's disease and Parkinson's disease. *Journal of the neurological sciences.* 2016;369:278-282.

59. Weamer EA, DeMichele-Sweet MA, Cloonan YK, Lopez OL, Sweet RA. Incident Psychosis in Subjects With Mild Cognitive Impairment or Alzheimer's Disease. *The Journal of clinical psychiatry.* 2016;77(12):e1564-e1569.

60. Yoon B, Yang DW, Hong YJ, et al. Differences in Depressive Patterns According to Disease Severityin Early-Onset Alzheimer's Disease. *Journal of Alzheimer's disease : JAD.* 2016;52(1):91-99.

61. Kahle-Wrobleski K, Andrews JS, Belger M, et al. Dependence Levels as Interim Clinical Milestones Along the Continuum of Alzheimer's Disease: 18-Month Results from the GERAS Observational Study. *The journal of prevention of Alzheimer's disease.* 2017;4(2):72-80.

62. Lethin C, Renom-Guiteras A, Zwakhalen S, et al. Psychological well-being over time among informal caregivers caring for persons with dementia living at home. *Aging & mental health.* 2017;21(11):1138-1146.

63. Mueller C, Huntley J, Stubbs B, et al. Associations of Neuropsychiatric Symptoms and Antidepressant Prescription with Survival in Alzheimer's Disease. *Journal of the American Medical Directors Association.* 2017;18(12):1076-1081.

64. Ritter AR, Leger GC, Miller JB, Banks SJ. Neuropsychological Testing in Pathologically Verified Alzheimer Disease and Frontotemporal Dementia: How Well Do the Uniform Data Set Measures Differentiate Between Diseases? *Alzheimer disease and associated disorders.* 2017;31(3):187-191.

65. Yoon B, Shim YS, Hong YJ, et al. Anosognosia and Its Relation to Psychiatric Symptoms in Early-Onset Alzheimer Disease. *Journal of geriatric psychiatry and neurology.* 2017;30(3):170-177.

66. Arthur PB, Gitlin LN, Kairalla JA, Mann WC. Relationship between the number of behavioral symptoms in dementia and caregiver distress: What is the tipping point? *International psychogeriatrics.* 2018;30(8):1099-1107.

67. Connors MH, Seeher KM, Crawford J, Ames D, Woodward M, Brodaty H. The stability of neuropsychiatric subsyndromes in Alzheimer's disease. *Alzheimer's & dementia : the journal of the Alzheimer's Association.* 2018;14(7):880-888.

68. Hager K, Henneges C, Schneider E, Lieb M, Kraemer S. [Alzheimer dementia: course and burden on caregivers : Data over 18 months from German participants of the GERAS study]. *Der Nervenarzt.* 2018;89(4):431-442.

69. Römhild J, Fleischer S, Meyer G, et al. Inter-rater agreement of the Quality of Life-Alzheimer's Disease (QoL-AD) self-rating and proxy rating scale: secondary analysis of RightTimePlaceCare data. *The mental health clinician.* 2018;16(1):131.

70. Siafarikas N, Selbaek G, Fladby T, Šaltytė Benth J, Auning E, Aarsland D. Frequency and subgroups of neuropsychiatric symptoms in mild cognitive impairment and different stages of dementia in Alzheimer's disease. *International psychogeriatrics.* 2018;30(1):103-113.

71. Yamakawa M, Lai AX, Kaup AR, Yaffe K, Byers AL. High Occurrence of Psychiatric Disorders and Suicidal Behavior Across Dementia Subtypes. *Psychogeriatrics : the official journal of the Japanese Psychogeriatric Society.* 2018;26(12):1191-1201.

72. Bauman J, Gibbons LE, Moore M, et al. Associations Between Depression, Traumatic Brain Injury, and Cognitively-Defined Late-Onset Alzheimer's Disease Subgroups. *Journal of Alzheimer's disease : JAD.* 2019;70(2):611-619.

73. Black BS, Johnston D, Leoutsakos J, et al. Unmet needs in community-living persons with dementia are common, often non-medical and related to patient and caregiver characteristics. *International psychogeriatrics.* 2019;31(11):1643-1654.

74. Borges LG, Rademaker AW, Bigio EH, Mesulam MM, Weintraub S. Apathy and Disinhibition Related to Neuropathology in Amnestic Versus Behavioral Dementias. *American journal of Alzheimer's disease and other dementias.* 2019;34(5):337-343.

75. Nunes PV, Schwarzer MC, Leite REP, et al. Neuropsychiatric Inventory in Community-Dwelling Older Adults with Mild Cognitive Impairment and Dementia. *Journal of Alzheimer's disease : JAD.* 2019;68(2):669-678.

76. Park S, Kim DK, Myung W, et al. Risk Factors of Behavioral and Psychological Symptoms in Patients with Alzheimer Disease: The Clinical Research of Dementia of South Korea Study. *Korean journal of family medicine.* 2019;40(1):16-21.

77. Cosseddu M, Benussi A, Gazzina S, et al. Progression of behavioural disturbances in frontotemporal dementia: a longitudinal observational study. *European journal of neurology.* 2020;27(2):265-272.

78. Huang MF, Lee WJ, Yeh YC, et al. Genetics of neuropsychiatric symptoms in patients with Alzheimer's disease: A 1-year follow-up study. *Journal of geriatric psychiatry and neurology.* 2020;74(12):645-651.

79. Liu C, Liu S, Wang X, Ji Y. Neuropsychiatric profiles in mild cognitive impairment with Lewy bodies. *Irish journal of medical science.* 2020:1-7.

**9) the number of dementia patients among the included participants is unclear: 2**

1. O'Donnell MJ, Lewis DL, Dubois S, Standish TI, Bédard M, Molloy DW. Behavioural and psychological symptoms in community-dwelling elderly persons with cognitive impairment and dementia: Prevalence and factor analysis. *Clinical gerontologist.* 2007;30(3):41-52.

2. Lövheim H, Sandman PO, Karlsson S, Gustafson Y. Behavioral and psychological symptoms of dementia in relation to level of cognitive impairment. *International psychogeriatrics.* 2008;20(4):777-789.

**10) for patients already with BPSD only: 2**

1. Nagata T, Shinagawa S, Nakajima S, et al. Classification of Neuropsychiatric Symptoms Requiring Antipsychotic Treatment in Patients with Alzheimer's Disease: Analysis of the CATIE-AD Study. *Journal of Alzheimer's disease : JAD.* 2016;50(3):839-845.

2. Nagata T, Nakajima S, Shinagawa S, et al. Baseline Predictors of Antipsychotic Treatment Continuation and Response at Week 8 in Patients with Alzheimer's Disease with Psychosis or Aggressive Symptoms: An Analysis of the CATIE-AD Study. *Journal of Alzheimer's disease : JAD.* 2017;60(1):263-272.

**11) using same data: 1**

1. Arbus C, Soto ME, Andrieu S, et al. The prevalence of clinically significant depressive symptoms in Alzheimer's disease: relationship with other psychological and behavioural symptoms. *International journal of geriatric psychiatry.* 2008;23(11):1209-1211.

**12) the definition of the reported symptoms of BPSD is ambiguous: 1**

1. Wulff J, Fänge AM. Self-reported symptoms of depression and anxiety among informal caregivers of persons with dementia: a cross-sectional comparative study between Sweden and Italy. 2020;20(1):1114.

**13) unavailable the full-text: 5**

1. Vilalta-Franch J. Non-cognitive aspects of dementia. *Revista de neurologia.* 1998;27(157):409-414.

2. Cortes F, Gillette-Guyonnet S, Nourhashemi F, Andrieu S, Cantet C, Vellas B. Recent data on the natural history of Alzheimer's disease: results from the REAL.FR Study. *The journal of nutrition, health & aging.* 2005;9(2):86-93.

3. Nourhashemi F, Amouyal-Barkate K, Gillette-Guyonnet S, Cantet C, Vellas B. Living alone with Alzheimer's disease: cross-sectional and longitudinal analysis in the REAL.FR Study. *The journal of nutrition, health & aging.* 2005;9(2):117-120.

4. Shih CS, Yan SH, Ho YH, Lin YT, Li JY, Lo YK. Psychiatric morbidity in dementia patients in a neurology-based memory clinic. *Acta neurologica Taiwanica.* 2005;14(4):179-186.

5. Xie HG, Wang LN, Yu X, et al. Incidence of disturbance in sleep behavior and its related factors in dementia patients and population of community. *Chinese Journal of Clinical Rehabilitation.* 2005;9(44):32-35.

**Supplement material 4. Raw data of each meta-analysis**

**1. Delusions**

1) Subgroup analysis: dementia type

1-1) Subgroup analysis: dementia type – Sensitivity analysis (removing outlier)

removing Rountree 2012

2) Subgroup analysis: dementia severity

2-1) Subgroup analysis: dementia severity – Sensitivity analysis (removing outlier)

removing Rountree 2012, Holmstrand 2020

3) Subgroup analysis: age of participants

3-1) Subgroup analysis: age of participants – Sensitivity analysis (removing outlier)

removing Rountree 2012, Holmstrand 2020

**2. Hallucinations**

1) Subgroup analysis: dementia type

1-1) Subgroup analysis: dementia type – Sensitivity analysis (removing outlier)

removing Wilson 2005, Rountree 2012, Holmstrand 2020

2) Subgroup analysis: dementia severity

2-1) Subgroup analysis: dementia severity – Sensitivity analysis (removing outlier)

removing Wilson 2005, Rountree 2012, Holmstrand 2020

3) Subgroup analysis: age of participants

3-1) Subgroup analysis: age of participants – Sensitivity analysis (removing outlier)

removing Rountree 2012, Holmstrand 2020

**3. Depression**

1) Subgroup analysis: dementia type

1-1) Subgroup analysis: dementia type – Sensitivity analysis (removing outlier)

removing Teri 1999

2) Subgroup analysis: dementia severity

2-1) Subgroup analysis: dementia severity – Sensitivity analysis (removing outlier)

removing Teri 1999

3) Subgroup analysis: age of participants

3-1) Subgroup analysis: age of participants – Sensitivity analysis (removing outlier)

removing Teri 1999

**4. Anxiety**

1) Subgroup analysis: dementia type

1-1) Subgroup analysis: dementia type – Sensitivity analysis (removing outlier)

removing Teri 1999

2) Subgroup analysis: dementia severity

2-1) Subgroup analysis: dementia severity – Sensitivity analysis (removing outlier)

removing Teri 1999

3) Subgroup analysis: age of participants

3-1) Subgroup analysis: age of participants – Sensitivity analysis (removing outlier)

removing Teri 1999

**5. Irritability**

1) Subgroup analysis: dementia type

2) Subgroup analysis: dementia severity

3) Subgroup analysis: age of participants

**6. Apathy**

1) Subgroup analysis: dementia type

2) Subgroup analysis: dementia severity

3) Subgroup analysis: age of participants

**7. Elation & mania**

1) Subgroup analysis: dementia type

2) Subgroup analysis: dementia severity

3) Subgroup analysis: age of participants

**8. Agitation & aggression**

1) Subgroup analysis: dementia type

2) Subgroup analysis: dementia severity

3) Subgroup analysis: age of participants

**9. Disinhibition**

1) Subgroup analysis: dementia type

1-1) Subgroup analysis: dementia type – Sensitivity analysis (removing outlier)

removing Peters 2006, Holmstrand 2020

2) Subgroup analysis: dementia severity

2-1) Subgroup analysis: dementia severity – Sensitivity analysis (removing outlier)

removing Peters 2006, Holmstrand 2020

3) Subgroup analysis: age of participants

3-1) Subgroup analysis: age of participants – Sensitivity analysis (removing outlier)

removing Peters 2006, Holmstrand 2020

**10. Aberrant motor behavior**

1) Subgroup analysis: dementia type

1-1) Subgroup analysis: dementia type – Sensitivity analysis (removing outlier)

removing Peters 2006, Holmstrand 2020

2) Subgroup analysis: dementia severity

3) Subgroup analysis: age of participants

3-1) Subgroup analysis: age of participants – Sensitivity analysis (removing outlier)

removing Peters 2006, Holmstrand 2020

**11. Wandering**

1) Subgroup analysis: dementia type

2) Subgroup analysis: dementia severity

3) Subgroup analysis: age of participants

**12. Eating disorder**

1) Subgroup analysis: dementia type

2) Subgroup analysis: dementia severity

3) Subgroup analysis: age of participants

**13. Sleep disorders**

1) Subgroup analysis: dementia type

1-1) Subgroup analysis: dementia type – Sensitivity analysis (removing outlier)

removing Holmstrand 2020

2) Subgroup analysis: dementia severity

2-1) Subgroup analysis: dementia severity – Sensitivity analysis (removing outlier)

removing Holmstrand 2020

3) Subgroup analysis: age of participants

3-1) Subgroup analysis: age of participants – Sensitivity analysis (removing outlier)

removing Holmstrand 2020
